# Mitochondrial Modulation as a Therapeutic Entry Point in Neurodegeneration

**DOI:** 10.3390/cells15181683

**Published:** 2026-09-17

**Authors:** Mateusz Mamala, Izabela Skowron, Karolina Marczuk, Arkadiusz Bujas, Jolanta Harasiuk, Julita Kulbacka

**Affiliations:** 1 Faculty of Medicine, Wroclaw Medical University, 50-367 Wroclaw, Poland; mateusz.mamala@student.umw.edu.pl (M.M.); karolina.marczuk@student.umw.edu.pl (K.M.); 2 The Students’ Research Group, SKN No. 148, 50-556 Wroclaw, Poland; izabela.klara.skowron@gmail.com (I.S.); a.bujas@umw.edu.pl (A.B.); 3 Faculty of Health Sciences, Wroclaw Medical University, 50-367 Wroclaw, Poland; 4 Department of Molecular and Cellular Biology, Faculty of Pharmacy, Wroclaw Medical University, 50-556 Wroclaw, Poland; jolanta.harasiuk@umw.edu.pl; 5 Department of Immunology and Bioelectrochemistry, State Research Institute Centre for Innovative Medicine, LT-08406 Vilnius, Lithuania

**Keywords:** mitochondrial dysfunction, neurodegeneration, mitophagy, oxidative stress, mitochondrial DNA, redox biomarkers, mitochondrial therapeutics

## Abstract

**Highlights:**

**What are the main findings?**

- The pathogenic and therapeutic significance of mitochondrial dysfunction varies across disorders and disease stages, acting as a pathway-proximal driver in some contexts and an amplifier of neuronal vulnerability in others.
- Broad antioxidant approaches have generally failed to modify clinical progression, whereas mechanism-specific strategies may offer greater translational potential in biologically defined populations.

**What are the implications of the main findings?**

- Mitochondria-directed therapies require mechanism-informed trial design, biomarker-enriched patient selection, and disease-stage-appropriate treatment timing.
- Future trials should distinguish pharmacodynamic target engagement from clinical benefit and evaluate mitochondrial modulation primarily as a precision-guided or adjunctive strategy.

**Abstract:**

Mitochondrial dysfunction is a recurrent but context-dependent feature of neurodegenerative disease, and its position in the pathogenic cascade differs fundamentally between disorders. This narrative review argues that this heterogeneity, rather than mitochondrial biology itself, determines therapeutic tractability. We synthesize evidence on mitochondrial regulation of neuronal development, organelle quality control, redox signaling and neuroinflammation; on oxidative biomarkers, whose clinical use remains constrained by limited disease specificity, methodological heterogeneity and insufficient longitudinal validation; and on therapeutic strategies ranging from antioxidants and NAD+ augmentation to mitochondrial genome engineering, targeted delivery and organelle transfer. A consistent pattern emerges across these domains: broadly acting interventions have repeatedly failed in sporadic disease, whereas the strongest translational signals arise where mitochondrial dysfunction is genetically anchored and pathway-proximal. Mitochondrial modulation is therefore unlikely to provide a universal disease-modifying strategy, but remains a useful, carefully targeted addition for patient groups identified by genetic or specific biological markers.

## 1. Introduction

Neurodegenerative disorders comprise a clinically and biologically heterogeneous group of conditions characterized by progressive dysfunction and loss of selectively vulnerable neuronal populations. Although their initiating mechanisms differ, mitochondrial abnormalities have been repeatedly described in Alzheimer’s disease, Parkinson’s disease, amyotrophic lateral sclerosis, Huntington’s disease, and inherited mitochondrial syndromes [1,2]. However, mitochondrial impairment does not occupy the same position in the pathogenic cascade of every disorder. In genetically defined mitochondrial diseases and selected defects of mitochondrial quality control, it may be closely linked to disease initiation, whereas in common sporadic disorders, it may develop alongside or downstream of protein aggregation, impaired proteostasis, inflammation, and other cellular stressors and subsequently amplify neuronal injury [1,2,3]. The relevant translational question is therefore not simply whether mitochondria are altered, but whether the observed dysfunction is sufficiently causal, measurable, and reversible to constitute a viable therapeutic target [1,2].

Neurons are particularly dependent on mitochondrial integrity because they have high energy demands, marked structural polarization, and limited regenerative capacity. Mitochondrial ATP production supports membrane excitability, ion-gradient restoration, axonal transport, synaptic vesicle cycling, and neurotransmission [4,5,6]. Beyond bioenergetics, mitochondria contribute to calcium buffering, redox signaling, metabolic adaptation, and the regulation of cell-death pathways [7,8,9]. Their importance begins before neuronal maturity. Differentiation of neural progenitor cells is accompanied by increased mitochondrial biogenesis, structural remodeling, and a shift from predominantly glycolytic metabolism toward greater reliance on oxidative phosphorylation [10,11,12]. Experimental modulation of mitochondrial metabolism can also alter the species-specific tempo of cortical neuronal maturation [13]. In the adult hippocampus, mitochondrial metabolism and dynamics contribute to neural stem-cell lineage progression and the functional integration of newly generated neurons [14,15].

Mitochondrial homeostasis is maintained by an interconnected quality-control network that includes biogenesis, fission and fusion, organelle transport, mitophagy, and preservation of mitochondrial DNA integrity [16,17,18,19]. Mitochondria also communicate directly with other organelles. Endoplasmic reticulum–mitochondria contact sites regulate calcium and lipid exchange and contribute to synaptic function [20,21,22,23,24]. Mitochondria–lysosome contacts participate in mitochondrial fission and organelle quality control, while their dysregulation has been demonstrated in neuronal models relevant to Parkinson’s disease [23,24]. Damaged mitochondria may additionally release mitochondrial DNA and other mitochondria-derived damage-associated molecular patterns that activate innate immune pathways and reinforce neuroinflammation [25,26,27]. These bidirectional interactions mean that mitochondrial dysfunction can be both a contributor to and a consequence of neuronal injury, with its importance varying according to disease, cell type, and stage [28].

Redox imbalance is among the most frequently investigated consequences of mitochondrial dysfunction. At physiological concentrations, mitochondrial reactive oxygen species participate in calcium regulation, synaptic plasticity, metabolic adaptation, and developmental signaling [9,29]. When reactive-species production exceeds antioxidant and repair capacity, oxidative modifications of lipids, proteins, and nucleic acids may accumulate and contribute to cellular dysfunction [30]. Lipid peroxidation products, protein oxidation markers, oxidative DNA damage products, and measures of antioxidant defense are therefore widely used as indirect indicators of oxidative stress [31]. Their interpretation is nevertheless affected by sample type, pre-analytical handling, analytical platform, age, diet, comorbidities, medication exposure, and concurrent inflammation [32]. Most oxidative biomarkers also lack sufficient disease specificity and longitudinal validation to function as independent diagnostic or prognostic tools in neurodegenerative disease [33]. They are currently more defensible as indicators of biological stress, components of multimarker panels, or pharmacodynamic readouts than as stand-alone clinical biomarkers.

The clinical experience with mitochondria-oriented interventions further demonstrates the importance of disease context. Randomized trials of MitoQ, high-dose coenzyme Q10, and inosine-mediated urate elevation—respectively, a triphenylphosphonium-conjugated ubiquinone that accumulates in the mitochondrial matrix, an endogenous electron carrier between complexes I/II and III that also acts as a lipophilic chain-breaking antioxidant, and a urate precursor intended to scavenge peroxynitrite—did not demonstrate slowing of clinical progression in early Parkinson’s disease [34,35]. In contrast, the nonrandomized, natural-history-controlled LEROS study reported treatment-associated visual benefit with idebenone, a short-chain quinone that transfers electrons directly to complex III and thereby bypasses a defective complex I, in Leber hereditary optic neuropathy, a genetically defined disorder in which mitochondrial respiratory-chain dysfunction is directly linked to disease biology [2,36]. This contrast is consistent with, but does not by itself prove, the hypothesis that mitochondrial therapies may be more effective when the molecular defect is pathway-proximal, and the treated population is biologically well defined [1,2]. Other interventions have demonstrated target engagement without established clinical efficacy. The phase I NADPARK trial showed that nicotinamide riboside can engage cerebral NAD+ metabolism in Parkinson’s disease [37], while a phase II resveratrol trial in Alzheimer’s disease demonstrated tolerability and changes in selected biomarkers without establishing clinical benefit [38]. Approaches targeting mitophagy, mitochondrial dynamics, mitochondrial genome maintenance, organelle-specific delivery, and mitochondrial transfer remain supported predominantly by preclinical or early translational evidence [39,40,41].

Accordingly, this narrative review examines mitochondrial regulation across neuronal development, functional maintenance, and neurodegenerative disease, with particular emphasis on mitochondrial quality control, genome stability, organelle dynamics and inter-organelle communication, redox imbalance, and neuroinflammation. It also evaluates oxidative and mitochondrial biomarkers and critically appraises current and emerging mitochondria-directed interventions, including their evidentiary maturity and translational limitations. By integrating developmental, mechanistic, biomarker, and therapeutic evidence, this review aims to clarify when mitochondrial modulation may represent a viable therapeutic entry point and why precision-guided or adjunctive applications are likely to be more credible than universal mitochondrial targeting.

## 2. Methods

This narrative review was based on a non-systematic literature search performed using the PubMed/MEDLINE and Google Scholar databases. Search terms combined concepts related to mitochondrial biology, including “mitochondrial dysfunction”, “mitophagy”, “mitochondrial dynamics”, “mitochondrial DNA”, “organelle communication”, “oxidative stress”, “redox signaling”, and “neuroinflammation”, with terms related to neurodevelopment, neurodegenerative diseases, biomarkers, and mitochondria-targeted therapies. Disease-related keywords included “Alzheimer’s disease”, “Parkinson’s disease”, “amyotrophic lateral sclerosis”, and “Huntington’s disease”, among others, together with broader terms encompassing neurodegenerative, neuroinflammatory, cerebrovascular, traumatic, mitochondrial, and neurogenetic disorders. Recent peer-reviewed studies were prioritized, while seminal publications were included to provide mechanistic and historical context. Human studies and clinical trials were preferentially considered when evaluating translational and therapeutic relevance. Additional publications were identified through manual screening of the reference lists of selected articles.

## 3. The Role of Mitochondria in Development and Functional Maintenance of the Nervous System

Mitochondria are central metabolic regulators that drive key neurological processes, from neurogenesis to plasticity, by integrating multiple signaling pathways. Their functions are tightly controlled by specific protein networks that adjust mitochondrial shape, number, and position to match changing demands in developing and mature neurons. Previous studies have shown that mitochondria are involved in various cell pathways related to the nervous system. For instance, recent studies suggest that neural stem cells possess increased mitochondrial DNA, controlled reactive oxygen species (ROS) levels, and morphological changes that enable them to switch from glycolysis to mitochondrial oxidative phosphorylation (OXPHOS), thereby driving proliferation and differentiation into neurons [42,43,44,45]. The metabolic shift is initiated by the upregulation of PGC-1α, a key orchestrator of mitochondrial biogenesis [10], which leads to enhanced synthesis of mitochondrial respiratory chain (MRC) complex subunits [46]. Mitochondrial mass expansion occurs to accommodate the MRC machinery, providing the oxidative phosphorylation capacity required to drive neuronal differentiation [11,47]. The metabolic transition to OXPHOS during neurogenesis is accompanied by mitochondrial biogenesis and structural reorganization, resulting in the elongated mitochondrial profiles characteristic of mature neurons [12,48,49].

Several studies suggest that neuronal maturation timing is intrinsically linked to metabolic output, with a direct correlation between the level of mitochondrial activity and the speed at which neurons reach maturity [13]. During the transition from neural stem/progenitor cells to neurons, cells undergo a conserved metabolic switch from glycolysis and fatty acid β-oxidation toward enhanced oxidative phosphorylation, accompanied by increased mitochondrial biogenesis, remodeling of morphology, and rising mitochondrial DNA content and reactive oxygen species, which together drive proliferation, fate commitment, and early differentiation [11,12,50,51].

In cortical neuron maturation, mitochondrial development and OXPHOS activity scale with species-specific developmental timing. Human neurons exhibit slower mitochondrial growth and lower respiratory activity than mouse neurons, and experimental stimulation of mitochondrial metabolism can accelerate human neuronal maturation and corticogenic temporal patterning, whereas inhibition slows mouse neuronal development [52,53]. It has been established that integrated multi-omics profiling of human iPSC-derived neurons reveals a crucial early phase of metabolic adaptation, characterized by expanding mitochondrial mass, elevated respiratory function, and upregulated antioxidant defenses, all while maintaining glycolytic flexibility [54].

From a detailed perspective, mitochondria help form new neurons by linking cellular energy use to changes in gene activity, such as histone acetylation, which uses acetyl-CoA. This process is further regulated by miR-124, which controls OXPHOS, and a unique RNA-level interaction, physical contact between mitochondria and the nucleus, facilitates the removal of nuclear RNAs, which triggers the transition into a differentiated state [52,53,55,56].

It has been established that mitochondria conduct neurulation, nucleic acid synthesis, and epigenetic modifications required for neural tube formation and cortical growth [57,58]. In the initial stage of neurodevelopment, mitochondria facilitate neural tube closure and cortical expansion by providing essential metabolites and one-carbon units that support epigenetic reconfiguration and rapid nucleic acid synthesis. This period of neurulation is marked by a metabolic transition, in which neural plate cells shift from a hypoxic, glycolytic profile to oxidative phosphorylation. This functional change is mirrored by structural adaptations in mitochondrial morphology and cristae organization as the neuroectoderm reorganizes into the neuroepithelium [58,59,60]. This developmental stage is characterized by a significant escalation in nucleic acid synthesis and global transcriptomic shifts. These processes are fundamentally driven by mitochondrial folate-mediated one-carbon metabolism, which provides the essential purine precursors and methyl groups required for the precise regulation of DNA and histone methylation patterns [60,61,62]. The upregulation of folate pathway genes, specifically the mitochondrial transporter SLC25A32, during neural tube closure shows the important role of mitochondrial one-carbon flow in neurulation. This necessity is further evidenced by the increased risk of neural tube defects associated with dietary folate deficiency and mutations in key folate-pathway enzymes [59]. In addition to folate metabolism, mitochondrial oxidative cycles govern neural stem cell fate and cortical progenitor maintenance by modulating NAD+/NADH ratios and acetyl-CoA availability. These metabolites serve as critical cofactors for histone acetylation, notably H3K27ac and H4K16ac, and sirtuin-mediated chromatin modifications, which ultimately involve the transition from self-renewal to neurogenesis [43,50,59,63].

Epigenetic modulators, including HDAC inhibitors, promote mitochondrial biogenesis and oxidative metabolism by activating NRF-1 and TFAM. The resulting expansion in mitochondrial mass and respiratory function is essential for supporting both neurogenesis and the maturation of neurons [64]. Prior research suggests that due to the high energy needs of the brain, neurons contain thousands of mitochondria to sustain excitability, ion gradients, and synaptic transmission [42,57].

**Figure 1:**
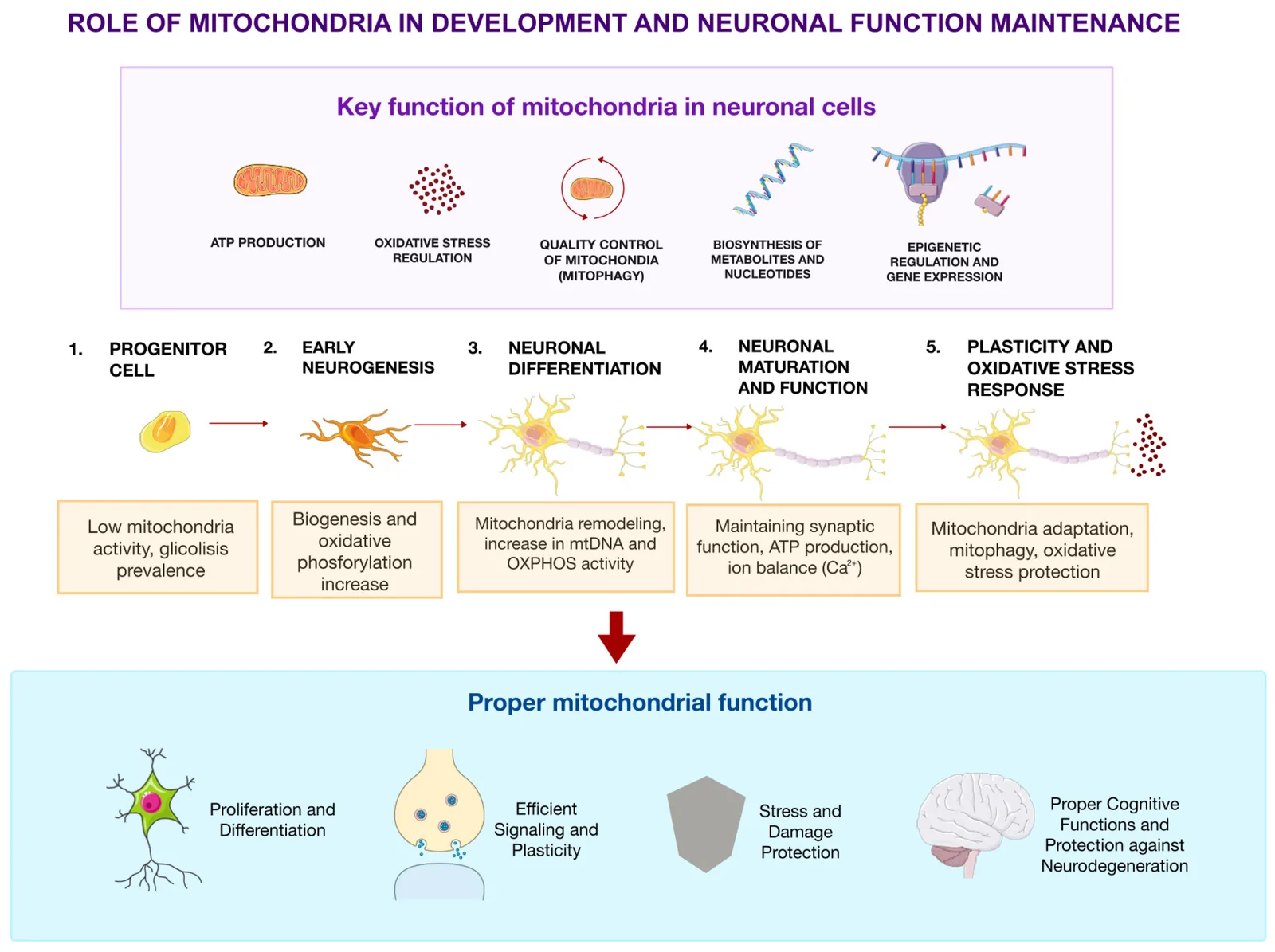
Role of mitochondrial contributions across neurodevelopment and mature neuronal function. OXPHOS, oxidative phosphorylation; mtDNA, mitochondrial DNA; ROS, reactive oxygen species; MRC, mitochondrial respiratory chain. Adapted from Servier Medical Art (https://smart.servier.com, accessed on 5 September 2026), licensed under CC BY 4.0 (https://creativecommons.org/licenses/by/4.0/, accessed on 5 September 2026). **🡓**—indicate direction of progression between stages and towards the resulting functional outcome; **🡒** horizontal arrows mark the sequential transition between developmental stages.

Synaptic transmission represents a significant metabolic demand, as ATP is essential in both pre- and postsynaptic compartments. Energy is consumed primarily to fuel the exo- and endocytic cycling of synaptic vesicles, neurotransmitter biosynthesis and loading, and the active extrusion of Ca^2+^ and Na^+^ ions following depolarization [4]. A few authors have recognized that mitochondria localize to axons, dendrites, and presynaptic terminals to power vesicle cycling and maintain Ca^2+^ balance, directly supporting synaptic plasticity, spine morphology, and axon branching [5,6,44,65]. Previous studies have emphasized the role of mitochondria in learning and memory through regulation of adult hippocampal neurogenesis [13]. The progression of the neural lineage depends on stage-specific mitochondrial processes, while proliferating progenitors rely on oxidative phosphorylation, maturing neurons require coordinated mitochondrial fission, fusion, and mitophagy for successful integration into hippocampal circuits [14,66]. Mitochondrial dysfunction, stemming from complex failure, loss of OPA1, or protein-folding stress, impairs NSC maintenance and differentiation. These defects ultimately manifest as reduced neurogenesis and hippocampus-dependent cognitive deficits (Figure 1), including spatial memory loss and neural hyperactivity [14,15,66].

## 4. Mitochondrial Dysfunction, Oxidative Stress and Their Contribution to the Pathogenesis of Neurodegenerative Disorders

**Figure 2:**
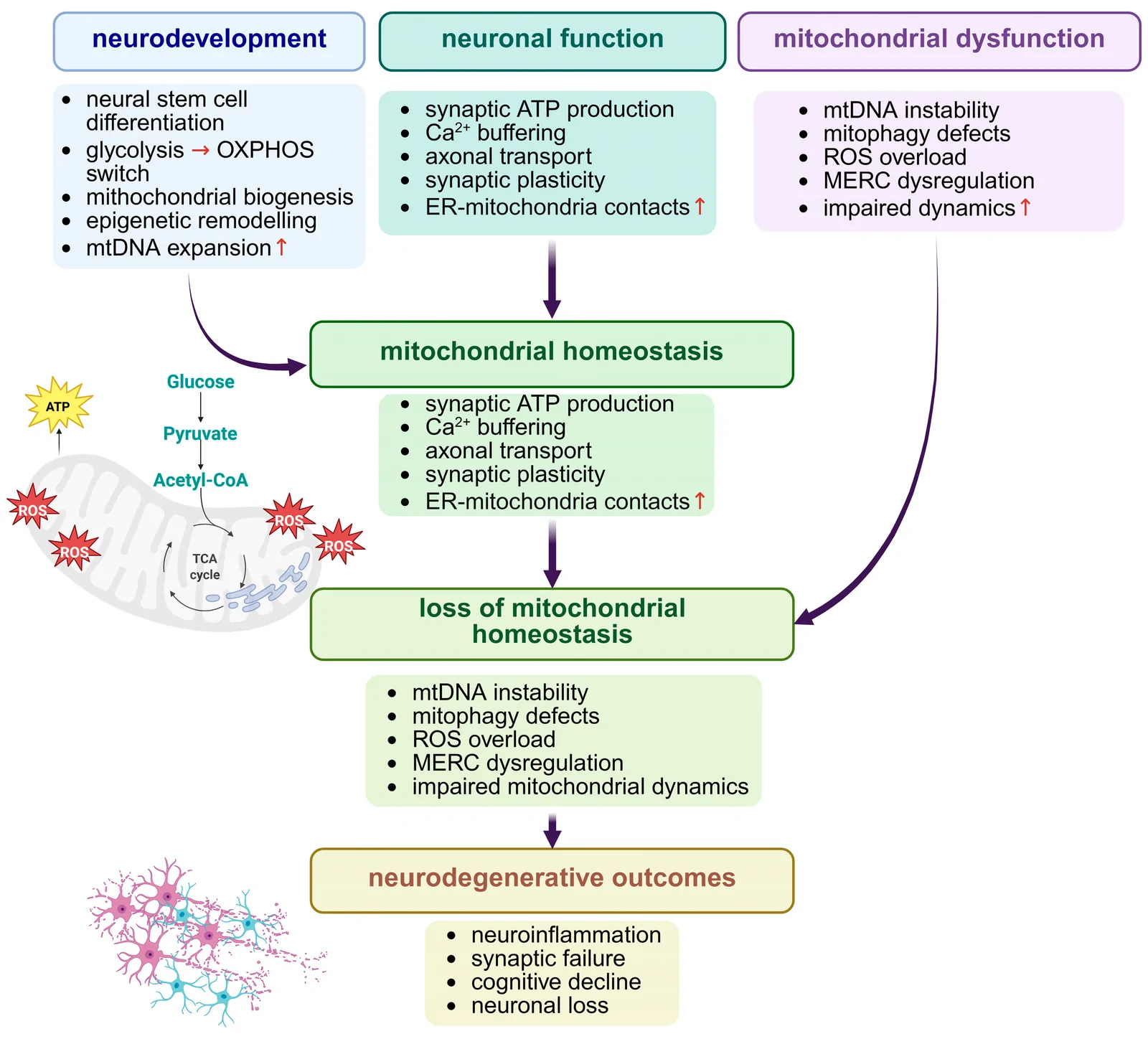
Mitochondrial homeostasis in relation to neurodevelopment, neuronal function, and neurodegenerative outcomes. MERC, mitochondria-endoplasmic reticulum contact; mtDNA, mitochondrial DNA; OXPHOS, oxidative phosphorylation; ROS, reactive oxygen species. Created in BioRender. Kulbacka, J. (2026) (https://BioRender.com/6rp5rgd, accessed on 5 September 2026). Red arrows indicate the direction of change in the indicated process (increase, or transition in the case of the glycolysis-to-OXPHOS switch).

Converging evidence indicates that mitochondrial complex I activity, mtDNA maintenance (TFAM), and finely tuned organelle dynamics (fission-fusion, transport, mitophagy) are all required for neural stem/progenitor proliferation, neuronal and oligodendroglial differentiation and survival; disruption at any of these levels leads to impaired neurogenesis, immature brain architecture, defective network activity, and cognitive decline [16,67,68]. Figure 2 shows how the demands of neurodevelopment and of mature neuronal function converge on mitochondrial homeostasis, defined by balanced fusion and fission, efficient oxidative phosphorylation, controlled ROS levels, Ca^2+^ signaling, and mitophagy-dependent quality control. When this balance is lost—through mtDNA instability, defective mitophagy, ROS overload, dysregulated mitochondria-endoplasmic reticulum contacts, or impaired dynamics—the consequences include neuroinflammation, synaptic failure, cognitive decline, and neuronal loss.

### 4.1. Mitophagy

Mitophagy is the selective autophagic degradation of damaged mitochondria, a process vital for preserving the integrity of the mitochondrial pool. Upon sensing stressors such as depolarization, the cell initiates mitophagy through either outer-membrane receptors or ubiquitin-mediated tagging. These signals trigger the engulfment of the organelle by autophagosomes and its eventual lysosomal proteolysis [69,70]. The literature review shows that the PINK1-Parkin axis is the primary mechanism for mammalian mitophagy. PINK1 acts as a sensor of mitochondrial health, accumulating on the surface of depolarized organelles where import is compromised. Once stabilized, PINK1 phosphorylates and activates the E3 ligase Parkin, which ubiquitinates the organelle. These tags act as a scaffold for autophagy receptors, facilitating the sequestration of damaged mitochondria into mitophagosomes [17,71,72,73]. In addition to the PINK1-Parkin axis, several ubiquitin-independent, receptor-mediated pathways, along with vesicular routes such as mitochondria-derived vesicles (MDVs) and secretory autophagy, facilitate mitochondrial turnover. These mechanisms operate in parallel with, or serve as critical alternatives to, canonical autophagy components when they are compromised [71,74]. It has been established that mitophagy serves both as a ubiquitous housekeeping mechanism and a specialized developmental tool. Its programmed activation is vital for erythrocyte maturation, the elimination of sperm mitochondria, early embryonic development, and the orchestration of cell fate transitions [75]. Several authors have recognized that imbalances in mitophagy are linked to a wide array of diseases, ranging from cancer and aging to neurodegenerative disorders like Parkinson’s disease. As a result, targeting mitophagy with small-molecule modulators represents a major frontier in current drug discovery [18,75,76,77].

### 4.2. mtDNA Instability

Mitochondrial DNA (mtDNA) instability in the nervous system, manifesting as point mutations, large-scale deletions, or copy-number depletion, severely compromises oxidative phosphorylation and neuronal viability. Due to their high ATP dependence and post-mitotic nature, neurons are particularly susceptible because they cannot dilute mutant genomes through cell division. Consequently, pathogenic mutations often reach critical functional thresholds at relatively low levels of heteroplasmy [78]. Prior research suggests that mutations in nuclear genes involved in mtDNA maintenance, such as POLG, TWNK, and TFAM, lead to syndromes characterized by mtDNA depletion or deletions. The resulting encephalopathic and neuropsychiatric manifestations underscore how impaired replication and repair pathways destabilize mitochondrial DNA in the central nervous system [19,79,80]. Recent studies suggest that defective mtDNA homeostasis is a common hallmark of aging and various neurodegenerative conditions, including Parkinson’s disease, Alzheimer’s disease, amyotrophic lateral sclerosis, and progressive external ophthalmoplegia, in which somatic mtDNA deletions, reduced copy number, or POLG-related replication defects have been documented in affected neuronal populations. By identifying somatic mtDNA deletions and copy-number fluctuations in specific cell types, most notably dopaminergic and pontine neurons, researchers have highlighted a critical link between mtDNA instability and the selective patterns of neuronal loss and diverse clinical phenotypes seen in the disorders [19,81,82,83]. Studies utilizing experimental models of neuronal mtDNA damage or pathogenic mutations have established mitochondrial genomic instability as a primary driver of circuit failure. These models exhibit a cascade of pathologies, including compromised mitochondrial transcription, diminished respiratory capacity, and irregular organelle disturbance. Such cellular defects manifest as synaptic impairment and cognitive decline, suggesting that mtDNA mutations are not merely secondary markers of aging, but active contributors to brain dysfunction [16,84,85,86].

### 4.3. Redox Signaling

Mitochondria function as essential redox signaling centers within the nervous system, linking oxidative metabolism to key processes such as neuronal plasticity, functional activity, and disease susceptibility. As a byproduct of oxidative phosphorylation, the mitochondrial electron transport chain and matrix dehydrogenases continuously generate reactive oxygen species, primarily superoxide and hydrogen peroxide, at rates determined by the cell’s metabolic activity and respiratory state [7,8]. Mitochondrial-derived ROS act as signaling molecules at physiological concentrations, regulating Ca^2+^ dynamics and the function of redox-sensitive proteins and ion channels. This redox signaling is fundamental to the orchestration of synaptic plasticity and neurogenesis, as well as the structural remodeling of circuits during critical periods of development [20,87,88]. Redox signaling is modulated by a sophisticated network of antioxidant enzymes, including MnSOD, glutathione peroxidase, catalase, and peroxiredoxins, supported by the non-enzymatic thiol antioxidant glutathione, which serves as the reducing substrate for glutathione peroxidase and is further refined by cell-specific metabolic profiles. While neurons depend primarily on oxidative phosphorylation for their energetic needs, astrocytes rely predominantly on glycolysis and possess a more robust antioxidant infrastructure to maintain homeostasis [8,9,29]. The available data show that a breakdown in redox homeostasis, stemming from mitochondrial ROS surges or faulty Ca^2+^ crosstalk, converts essential signaling molecules into toxic oxidative agents. The resulting oxidative stress compromises cellular integrity through macromolecular damage and mitochondrial pore activation, driving the apoptotic and necrotic pathways linked to ischemic and neurodegenerative conditions [8,29,30].

### 4.4. ER-Mitochondria Contacts

Endoplasmic Reticulum (ER)-mitochondria contacts, often organized into mitochondria-associated membranes (MAMs) or MERCS, are abundant throughout neurons, in somata, dendrites, axons and synapses and form nanometer-scale junctions that enable rapid exchange of calcium, lipids and metabolic signals between the two organelles [21,87]. At these sites, calcium release from the ER is efficiently taken up by mitochondria via specialized protein complexes, tightly coupling Ca^2+^ signaling to ATP production, shaping synaptic Ca^2+^ transients and supporting neurotransmission and plasticity [21,22,88]. MERCs coordinate lipid metabolism and mitochondrial dynamics, alongside autophagy and redox signaling. These integrated functions enable MERCs to act as metabolic sensors that determine neuronal survival or apoptosis in response to physiological and pathological stress. Excessive transfer at these sites produces mitochondrial calcium overload, a mechanism of particular importance in neurodegeneration. Sustained matrix Ca^2+^ accumulation stimulates dehydrogenase activity and superoxide generation, lowers the threshold for opening of the mitochondrial permeability transition pore, and dissipates the membrane potential, releasing cytochrome c and committing the neuron to death. Calcium overload has been implicated in excitotoxic injury, in Huntington’s disease, where mutant huntingtin sensitizes mitochondria to Ca^2+^, and in Parkinson’s disease, where pacemaking activity imposes a continuous Ca^2+^ burden on dopaminergic neurons [20,21,87]. The VAPB-PTPIP51 tethering complex is essential for maintaining ER-mitochondria proximity at synaptic terminals. Although these contact sites are bolstered by neuronal activity, their loss in pathological contexts leads to a cascade of dysfunction, including defective Ca^2+^ signaling and bioenergetic failure, which manifests as altered spine morphology and impaired synaptic transmission [20,89]. Emerging evidence suggests that disrupted ER-mitochondria communication is a common denominator in various neurological and neurodevelopmental conditions. By appearing as early features in diseases like ALS and stroke, these contact sites represent a central nexus where degenerative and regenerative pathways converge, offering a novel frontier for the development of disease-modifying therapies [21,87,90].

### 4.5. Lysosome Contacts

Mitochondria-lysosome contacts are dynamic membrane contact sites that enable direct, non-fusing crosstalk between the two key organelles and have emerged as important regulators of neuronal homeostasis. High-resolution EM and super-resolution imaging show that ~15% of lysosomes are closely opposed to mitochondria at any time, with an intermembrane distance of ~10 nm and average tethering durations of ~60 s, sometimes extending to 13 min [24,91,92]. Unlike mitophagy or the formation of mitochondria-derived vesicles, these contact sites operate without the recruitment of autophagic machinery. Furthermore, they do not support the large-scale exchange of luminal materials, distinguishing them as platforms for signaling and metabolic crosstalk rather than organelle turnover [91,92,93]. These contact sites act as regulatory platforms for mitochondrial architecture, driving fission via a Rab7-dependent mechanism. Specifically, mitochondrial FIS1 recruits the Rab7 GTPase-activating protein TBC1D15 to the interface, where it modulates Rab7 GTPase cycling, thereby arranging mitochondrial network remodeling. In neurons, these interfaces are established throughout the soma, axons, and dendrites, where they serve as upstream regulators of mitochondrial distribution and physiology. Pathological mutations, such as those in GBA1 associated with Parkinson’s disease, are known to abnormally prolong contact duration, thereby destabilizing mitochondrial homeostasis [23,24,94]. Altered abundance or composition of mitochondria-lysosome contacts, including changes in tethering proteins such as MFN2, GDAP1, RAB7 and TBC1D15, is increasingly implicated in neurodegenerative and neurogenetic disorders, highlighting these nanometer-scale interfaces as critical nodes in organelle quality control and potential therapeutic targets [94,95,96].

### 4.6. Neuroinflammation

Mitochondrial dysfunction and neuroinflammation act as reciprocal drivers of neurodegenerative pathogenesis, establishing a self-amplifying vicious cycle in conditions such as Alzheimer’s, Parkinson’s, and ALS. Damaged mitochondria release mitochondrial-derived damage-associated molecular patterns (mtDAMPs), notably mtDNA, cardiolipin, ATP, and mitochondrial ROS, which trigger pattern-recognition receptors and inflammasomes, specifically NLRP3, within microglia and other glial cells. This activation culminates in the robust secretion of pro-inflammatory mediators, including IL-1β, IL-18, and TNF [25,26]. Chronic activation of the innate immune system acts as a persistent driver of neuronal death and mitochondrial collapse. This self-sustaining inflammatory state is a cornerstone of progressive neurodegeneration, linking initial organelle dysfunction to widespread circuit failure [28,97,98]. Mitochondrial dysfunction often precedes apparent neuroinflammation; initial defects in oxidative phosphorylation, Ca^2+^ homeostasis, and organelle dynamics prime the neuronal and microglial landscape for an exaggerated inflammatory response. These early disruptions in mitochondrial quality control, specifically mitophagy, establish a heightened state of sensitivity that exacerbates subsequent immune activation [27,28,57]. In turn, inflammatory cytokines and ROS inflict further damage on mtDNA, compromise respiratory chain integrity, and dysregulate mitochondrial quality control. This sustained assault deepens cellular energy failure and exacerbates oxidative stress, effectively entrenching the pathological cycle [25,98,99]. Across age-related neurodegenerative diseases, this bidirectional crosstalk positions mitochondria as an immune checkpoint linking metabolic stress to innate immune signaling, highlighting mtDAMP release, inflammasome activation, and mitochondrial quality-control pathways as promising therapeutic targets to interrupt the feed-forward loop of neuroinflammation and neurodegeneration [27,28,100,101].

## 5. Biomarkers of Free Radical-Mediated Damage in Neurodegenerative Diseases

**Figure 3:**
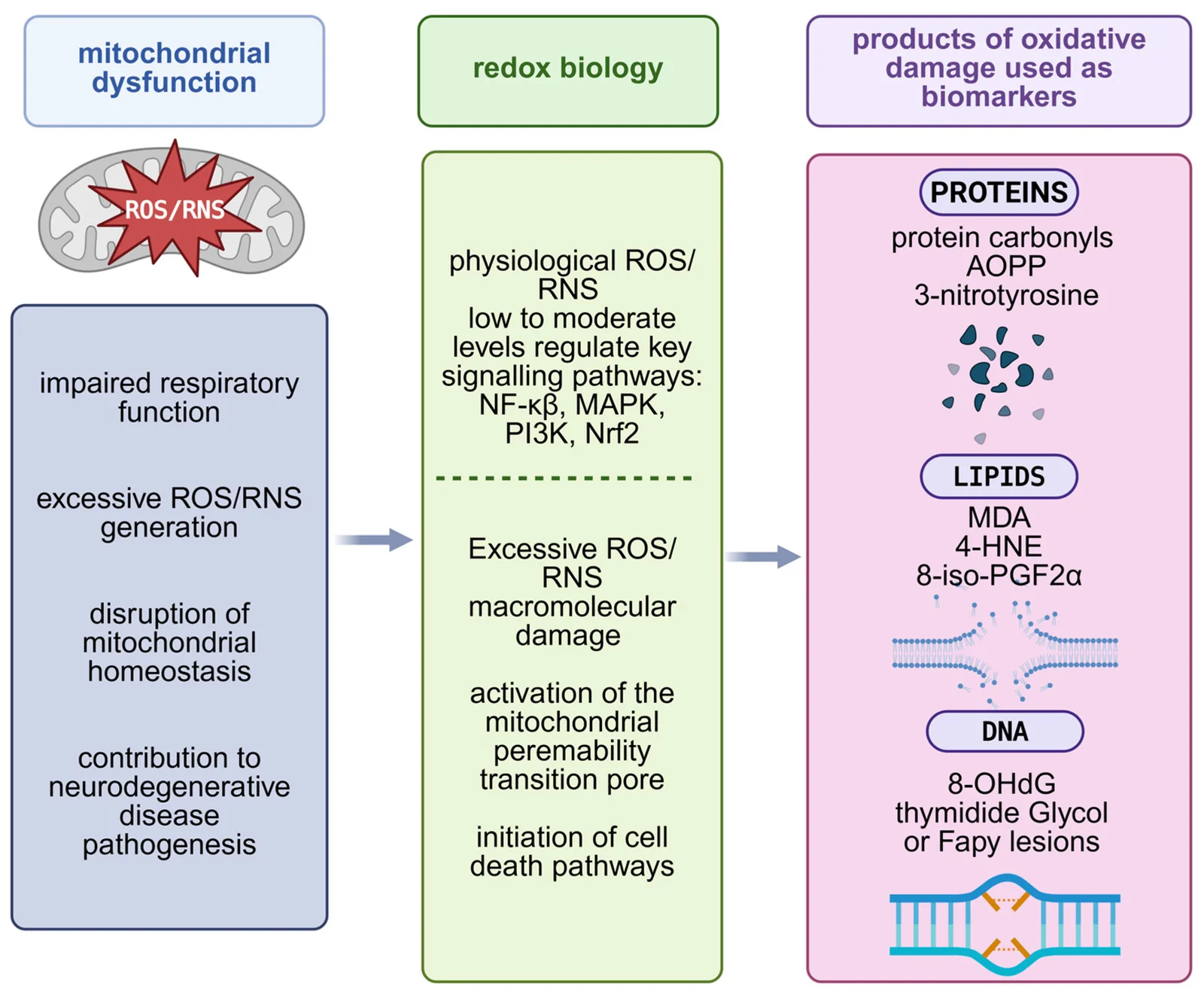
From mitochondrial dysfunction to oxidative biomarkers in neurodegeneration. Electron leak from the respiratory chain generates superoxide and hydrogen peroxide; when antioxidant capacity is exceeded, reactive species modify proteins (protein carbonyls, AOPP, 3-nitrotyrosine), lipids (MDA, 4-HNE, 8-iso-PGF2α, conjugated dienes) and DNA (8-OHdG, thymidine glycol, Fapy lesions). Because the radicals themselves are short-lived, human studies rely on these stable end products rather than on direct detection; quantification methods are summarized in Table 1. Sustained oxidative load also opens the mitochondrial permeability transition pore and initiates cell-death signaling. AOPP, advanced oxidation protein products; MDA, malondialdehyde; 4-HNE, 4-hydroxy-2-nonenal; 8-OHdG, 8-hydroxy-2′-deoxyguanosine. Created in BioRender. Kulbacka, J. (2026) https://BioRender.com/6rp5rgd.

Free radicals are atoms, molecules, or ions characterized by the presence of one or more unpaired electrons in their outer orbitals, which confers high chemical reactivity. In biological systems, the principal reactive species include ROS and reactive nitrogen species (RNS), both of which comprise free radical and non-radical species. Importantly, mitochondria are not the sole source of these species: NADPH oxidases (NOX), particularly NOX2 in activated microglia, together with xanthine oxidase, uncoupled nitric oxide synthase and peroxisomal oxidases, generate ROS at the plasma membrane and in other compartments. The presence of oxidative stress in nervous tissue therefore cannot be attributed to a mitochondrial defect by default, and source attribution requires compartment-specific evidence [102]. Physiological concentrations of oxygen- and nitrogen-derived reactive species, referred to as oxidative eustress, are characterized by low to moderate oxidant levels that regulate key biochemical reactions and redox-sensitive signaling pathways, including NF-κB, MAPK, PI3K, and Nrf2. In contrast, excessive ROS/RNS production from endogenous sources or exogenous factors leads to oxidative stress, a detrimental state associated with cellular damage [103]. Therefore, in biological systems, free radicals exert a dual function: they arise as potentially harmful by-products of aerobic metabolism that can induce oxidative damage and impair tissue function, while simultaneously acting as signaling mediators that initiate adaptive and beneficial cellular stress responses [104]. Free radicals can interact with a wide range of biomolecules in their surroundings, leading to molecular damage. Although excessive radical generation is detrimental to cellular integrity, this same property enables indirect assessment of radical burden through measurement of radical-induced biomolecular damage [105]. Mitochondrial dysfunction promotes excessive ROS generation, driving oxidative stress-mediated injury to cellular macromolecules and activation of the mitochondrial permeability transition pore. ROS overload disrupts mitochondrial homeostasis and initiates cell death pathways, ultimately contributing to the pathogenesis of diverse diseases. Because ROS/RNS are short-lived and highly reactive, direct assessment is limited. Most human studies therefore rely on stable downstream products of oxidative damage rather than on direct radical detection. This has major implications for biomarker development [106]. Although direct radical detection is mechanistically attractive, indirect markers are more practical for clinical use. However, oxidative readouts should not be treated as interchangeable. Protein oxidation, lipid peroxidation, oxidative DNA damage, and altered antioxidant defenses capture overlapping but distinct aspects of redox stress, and their value depends strongly on sample type, assay standardization, comorbidity, and disease stage. As a result, many oxidative biomarkers are better viewed as indicators of ongoing biological stress than as stand-alone diagnostic tools [31,32]. Figure 3 demonstrates the way from primary mitochondrial dysfunction to measurable oxidative biomarkers. Electron leak from the respiratory chain and matrix dehydrogenases generates superoxide and hydrogen peroxide; when antioxidant capacity is exceeded, reactive oxygen and nitrogen species modify proteins (protein carbonyls, advanced oxidation protein products, 3-nitrotyrosine), lipids (malondialdehyde, 4-hydroxy-2-nonenal, 8-iso-prostaglandin F2α, conjugated dienes) and DNA (8-hydroxy-2-deoxyguanosine, formamidopyrimidine lesions, strand breaks). Because the radicals themselves are short-lived, human studies rely on stable end products and antioxidant-enzyme readouts rather than on direct detection. Corresponding quantification methods and their limitations are also summarized in Table 1. ROS, reactive oxygen species; RNS, reactive nitrogen species.

### 5.1. Potential Markers in the Early Detection of Neurodegenerative Diseases

Oxidative stress is a central pathogenic mechanism in neurodegenerative disorders such as Alzheimer’s disease and Parkinson’s disease. Biomarkers of free radical-mediated damage constitute sensitive indicators of early neurodegenerative processes [107]. Nonetheless, they should not be described as validated early diagnostic tools. At present, their strongest potential lies in biological enrichment and preclinical risk stratification, especially when combined with disease-specific biomarker platforms rather than used alone. The most promising candidates are therefore those that combine mechanistic plausibility with relative stability, feasible sampling, and at least partial reproducibility across cohorts. On this basis, the markers reviewed here fall into three tiers of translational readiness. Tier 1, closest to application, comprises 8-OHdG and 8-iso-PGF2α, which are stable, measurable by standardized immunoassay or mass spectrometry in urine, plasma and CSF, and reproducible enough for cohort enrichment and pharmacodynamic monitoring. Tier 2 comprises protein carbonyls, AOPP, MDA and 4-HNE, which are mechanistically informative and widely reported but limited by assay heterogeneity and weak disease specificity. Tier 3 comprises EPR spin trapping, conjugated dienes and thymidine glycol, which remain research tools rather than deployable readouts. In our view, 8-OHdG and 8-iso-PGF2α carry the greatest near-term clinical potential, and only as components of multimarker panels alongside disease-specific biomarkers, never as stand-alone tests [108].

#### 5.1.1. Electron Paramagnetic Resonance (EPR)

Free radicals contain unpaired electrons, which drive their biochemical activity. Even though free radicals are highly reactive and short-lived, their direct detection is possible using Electron Paramagnetic Resonance (EPR) spin trapping, which stabilizes transient species as spin adducts for sensitive monitoring. EPR is technically demanding, difficult to standardize across centers, and poorly suited to large longitudinal neurodegeneration cohorts. It therefore remains more valuable as a mechanistic reference method than as a realistic near-term biomarker platform for routine clinical application [109].

#### 5.1.2. Indirect Assessment—Oxidative Modification of Biomolecules

Indirect assessment can be achieved through quantitative evaluation of oxidative modifications of biomolecules. Oxidative modifications of proteins (protein carbonyls, advanced oxidation protein products [AOPP]), lipids (MDA, 4-HNE, 8-iso-PGF2α), and DNA (8-OHdG, thymidine glycol) can be quantitatively assessed in biological fluids and tissues, providing translationally relevant readouts of redox imbalance. The integration of these biomarkers into clinical studies may enable preclinical detection and improved stratification of patients at risk for progressive neurodegeneration [110]. These damaged products can be quantified in accessible matrices and, in principle, may support risk stratification or response monitoring. However, their readiness for clinical translation is uneven. Some are analytically accessible but biologically nonspecific; others are mechanistically informative but too technically demanding for broad implementation. A more critical framework is therefore to prioritize markers by translational readiness, not only by biochemical plausibility. Consensus methodological guidance for measuring reactive species and oxidative damage should be applied when selecting, performing, and reporting these assays [111,112].

##### Oxidative Modifications of Proteins

Protein carbonylation arises either directly, through metal-catalyzed oxidative attack on lysine, arginine, proline, and threonine side chains, or indirectly, through Michael addition of reactive lipid-peroxidation aldehydes (4-HNE, MDA, acrolein) to nucleophilic lysine, histidine, and cysteine residues. It is a major oxidative stress outcome that functions in redox-sensitive signaling, regulating autophagy, proliferation, transcription, and apoptosis, and contributes to mitochondrial and endoplasmic reticulum dysfunction, linking oxidative stress to metabolic disease [113]. Protein carbonyl (PC) content is a widely used marker of protein oxidation, reflecting oxidative stress in clinical samples. PCs form via ROS-mediated oxidation of protein backbones and amino acid residues and can be quantified spectrophotometrically using DNPH, or detected via 2D gel electrophoresis, Western blot, or OxyBlot [111]. Advanced Oxidation Protein Products (AOPPs) are key biomarkers of oxidative stress because proteins, which are abundant in cells, plasma, and tissues, are major targets of free radicals. Levels increase in hypoxic newborns, particularly preterm infants, reflecting radical-mediated protein damage that amplifies tissue injury. AOPPs remain stable at −20 °C and −80 °C for six months, enabling batched spectrophotometric analysis calibrated with chloramine-T at 340 nm [114]. 3-Nitrotyrosine is a potential biomarker of oxidative stress, generated when reactive peroxynitrite nitrates both free and protein-bound tyrosine residues. This protein nitration at the subcellular level induces structural changes that disrupt the cytoskeleton, ultimately contributing to neurodegenerative processes [115]. GC-based approaches provide the most sensitive detection of 3-nitrotyrosine (3-NT), although they involve a lengthy preparatory derivatization process. HPLC does not need a derivatization step, although its accuracy is lower compared to GC [116]. Sulfur-containing amino acids, particularly cysteine and methionine, are highly prone to oxidative modifications. Cysteine undergoes extensive ROS-mediated post-translational changes, forming oxidized derivatives such as sulfenic, sulfinic, and sulfonic acids as well as inter- and intramolecular disulfides, while methionine is converted to the stable methionine sulfoxide. These oxidized forms can be readily detected in serum and plasma using HPLC or chromatographic methods coupled with MS, serving as sensitive biomarkers for the diagnosis and monitoring of oxidative stress-related human diseases [113]. Glycation end-products, including pentosidine and pyrraline, are protein modifications potentiated by oxidative stress via glycoxidation and serve as sensitive biomarkers of oxidative protein damage in human diseases. Chromatography-based methods, including HPLC and UPLC coupled primarily with MS/MS, offer the highest sensitivity for detecting glycoxidation products and are considered the gold standard for identifying both free and protein-bound forms. However, their routine use in clinical studies is limited, largely due to high costs [117].

##### Oxidative Modifications of Lipids

Malondialdehyde (MDA), a key lipid peroxidation product, is a widely used biomarker of oxidative stress in biological samples. Under acidic conditions, MDA reacts with thiobarbituric acid to form a chromophoric adduct that can be measured spectrophotometrically or fluorometrically, though cross-reactivity with other aldehydes may occur. More precise quantification is achieved using HPLC or GC-MS, which enable specific detection at the expense of more laborious sample preparation [110]. 8-Iso-prostaglandin F2α (8-iso-PGF2α), generated via nonenzymatic peroxidation of arachidonic acid, serves as a sensitive biomarker of lipid oxidative damage and can be quantified by UHPLC-MS/MS for clinical assessment of oxidative stress [118]. Conjugated dienes (CDs), formed during free radical-induced autoxidation of polyunsaturated fatty acids, serve as early and sensitive biomarkers of lipid peroxidation and can be quantified by their characteristic maximal UV absorption at 233 nm. 4-Hydroxy-2-nonenal (4-HNE), a highly reactive α,β-unsaturated hydroxyalkenal produced during lipid peroxidation, serves as a key biomarker of oxidative damage to cellular membranes and can be detected in tissues using immunohistochemistry or quantified by HPLC [119]. Lipid hydroperoxides (LOOH), the primary oxidation products of polyunsaturated fatty acids, represent early markers of lipid peroxidation and can be quantified using the ferrous oxidation-xylenol orange (FOX) assay, which detects LOOH through the formation of a colored ferric-xylenol orange complex [111]. Oxidized low-density lipoproteins (oxLDL) in serum or plasma are commonly used as biomarkers of oxidative stress and can be measured via a sandwich ELISA employing the monoclonal antibody 4E6, which targets a conformational epitope on oxidized ApoB-100 [120].

##### Oxidative Modification of DNA

8-Hydroxy-2-deoxyguanosine (8-OHdG) is a well-established biomarker of DNA oxidative damage. It is formed when hydroxyl radicals, highly reactive oxygen species, attack guanine bases in DNA, a process first described by Kasai and Nishimura in 1984. 8-OHdG can be measured in clinical samples using ELISA or immunohistochemistry, offering simpler and more practical alternatives to HPLC-ECD, while flow cytometry with OxyDNA-FITC provides additional quantitative assessment [121]. Thymidine glycol (Tg) is a DNA lesion generated under oxidative stress or ionizing radiation that inhibits nuclease P1 cleavage at the adjacent 3′ phosphodiester bond, resulting in the release of a dinucleotide containing the lesion. This dinucleotide can be accurately quantified using LC-MS/MS coupled with stable isotope-labeled standards, allowing sensitive and specific detection of Tg in DNA. Even though HPLC electrospray tandem mass spectrometry is highly effective for Tg analysis, its limited chromatographic resolution restricts its applicability for detecting oxidative damage in guanine, the most oxidation-prone DNA base [122]. Formamidopyrimidine (Fapy) lesions, DNA-protein crosslinks, and single- and double-strand breaks constitute the remaining classes of oxidative DNA damage. Fapy-dG is more mutagenic in mammalian systems than 8-oxodG and is therefore of biomarker interest, whereas crosslinks and strand breaks are chemically heterogeneous and strongly influenced by metabolic state and cell-cycle phase. All can be quantified, but by methods better suited to mechanistic work than to clinical cohorts, and none is currently deployable as a biomarker in neurodegenerative disease. The major biomarkers of oxidative damage and their quantification methods are summarized in Table 1.

**Table 1:** Biomarkers of Oxidative damage across biomolecules and their quantification methods.

Type of Biomolecule	Biomarker/Modification	Key Meaning	Quantitative Methods
Proteins	Protein carbonyls (PC)	ROS-driven protein oxidation; carbonylation involves Lys/His/Cys residues	DNPH spectrophotometry, 2D-gel, Western blot, OxyBlot [111]
Proteins	AOPP	Oxidative protein damage marker, linked to NOX/mitochondrial ROS amplification	Spectrophotometry (chloramine-T calibration, 340 nm) [114]
Proteins	3-Nitrotyrosine (3-NT)	Peroxynitrite-driven tyrosine nitration	GC (most sensitive, derivatization), HPLC (no derivatization, lower accuracy) [115,116]
Proteins	Oxidized Cys/Met	Oxidation of sulfur amino acids under ROS	HPLC, chromatography coupled with MS [113]
Proteins	Glycoxidation products (AGEs)	Oxidative-stress-potentiated protein glycoxidation	HPLC/UPLC–MS/MS (highest sensitivity, “gold standard”) [117]
Lipids	MDA	Lipid peroxidation product, TBA adduct, can be non-specific	TBARS (spectro/fluoro), HPLC, GC-MS [110,111]
Lipids	8-iso-PGF2α	Nonenzymatic arachidonic acid peroxidation marker	UHPLC-MS/MS [118]
Lipids	Conjugated dienes (CDs)	Early PUFA autoxidation (early lipid peroxidation)	UV absorbance 233 nm [111]
Lipids	4-HNE	Reactive lipid-peroxidation aldehyde, membrane damage marker	Immunohistochemistry, HPLC [119]
Lipids	LOOH	Primary PUFA oxidation products (early lipid peroxidation)	FOX assay (ferrous oxidation-xylenol orange) [111]
Lipids	oxLDL	Oxidatively modified LDL (oxidized ApoB-100 epitope)	Sandwich ELISA (mAb 4E6) [120]
DNA	8-OHdG	Hydroxyl radical attack on guanine (oxidative DNA damage)	ELISA, immunohistochemistry, flow cytometry (OxyDNA-FITC) [121,122]
DNA	Thymidine glycol (Tg)	Oxidative/radiation DNA lesion, quantified as lesion-containing dinucleotide	LC-MS/MS with stable isotope standards, HPLC-ESI-MS/MS (noted limitation) [122]
DNA	Fapy lesions (Fapy-dA/Fapy-dG)	Oxidative purine lesions, reduced polymerase fidelity, repair substrates	LC-MS/MS (gold standard; requires enzymatic DNA digestion), GC-MS (derivatization); tagging, fluorescence [122,123]
DNA	DNA–protein crosslinks (DPCs)	Covalent DNA-protein adducts (direct/indirect oxidative mechanisms)	Comet assay (indirect), filter retention, SDS/K^+^ precipitation, alkaline elution, gradient separation, proteomics for ID [124]
DNA	SSBs/DSBs	ROS-driven DNA breaks; clustered SSBs → DSBs (20–40 DSB/cell/Gy noted)	Comet assay, TUNEL, nick translation, STRIDE, imaging γH2AX/53BP1/XRCC1 [125]

→ Arrows denote conversion or transition between the indicated states.

#### 5.1.3. Indirect Assessment—Antioxidant Markers

Members of antioxidant defense systems are also widely applied as indirect biomarkers reflecting oxidative stress burden. These enzymatic antioxidants include superoxide dismutase (SOD), catalase (CAT), and glutathione peroxidase (GSH-Px) [126]. Their appeal lies in the fact that they reflect host response rather than injury accumulation alone. Their limitation is that they are strongly shaped by systemic physiology, adaptive compensation, diet, medication exposure, and comorbidity. They should therefore be interpreted as contextual markers of redox state, rather than as direct surrogates for neurodegeneration-related oxidative injury. In translational studies, they may add value when paired with damage markers, but rarely provide convincing stand-alone information [31].

#### 5.1.4. Positron Emission Tomography (PET)

PET-based imaging of reactive oxygen and nitrogen species is conceptually attractive because it enables spatially resolved redox imaging in the living brain. If sufficiently selective tracers can be validated, such methods could help bridge the gap between fluid biomarkers and regional disease biology. Recent probes illustrate both the promise and the constraints of this approach: [18F] fluoroedaravone reacts with a broad spectrum of reactive oxygen and nitrogen species, crosses the blood–brain barrier, and detects oxidative stress in murine models of stroke and tauopathy, but has not yet been validated in humans [127]. At present, however, the field remains early, and PET-based redox imaging should be regarded as an emerging translational direction rather than an established biomarker platform. Its future relevance will depend on tracer specificity, signal interpretability, and the ability to distinguish oxidative burden from broader inflammatory or metabolic activity [106].

### 5.2. The Role of Free Radical Markers in Neurodegenerative Diseases

Across neurodegenerative disorders, oxidative biomarkers are best understood as partially convergent indicators of biological stress rather than disease-specific signatures. Their repeated detection across diverse conditions reinforces the pathogenic relevance of redox imbalance, but also highlights a central limitation: the broader the applicability of a marker, the weaker its specificity for any single disease. This does not diminish their biological value, but it does constrain their clinical role. At present, the strongest use case for oxidative biomarkers is mechanistic enrichment, multimarker stratification and potentially pharmacodynamic monitoring, rather than stand-alone diagnosis. The strength of the underlying mitochondrial hypothesis also differs sharply between the disorders compared below. Genetic evidence anchors mitochondrial involvement causally in Parkinson’s disease with PINK1 or PRKN mutations, in Huntington’s disease, and in Friedreich ataxia, where frataxin deficiency directly impairs iron-sulfur cluster assembly and respiratory chain function. By contrast, in mild cognitive impairment, dementia with Lewy bodies, multiple system atrophy, progressive supranuclear palsy, corticobasal degeneration, multiple sclerosis, and Creutzfeldt–Jakob disease, comparable causal evidence is lacking, and mitochondrial abnormalities are more plausibly secondary to protein aggregation, neuroinflammation, or neuronal loss. Oxidative readouts in the latter group should accordingly be interpreted as markers of tissue stress rather than as evidence of a primary mitochondrial lesion [106]. Free radical-related biomarkers, sample types, translational relevance, and key limitations across neurodegenerative diseases are compared in Table 2.

#### 5.2.1. Alzheimer’s Disease (AD)

In Alzheimer’s disease, lipid peroxidation markers such as MDA, 4-HNE, and isoprostanes and oxidative DNA damage readouts such as 8-OHdG/8-oxodG are among the most consistently studied oxidative biomarkers across brain tissue, CSF, and peripheral biofluids. This makes AD one of the strongest disease contexts for oxidative biomarker research. However, even in AD, the evidence is stronger for biological association than for validated diagnostic or prognostic utility. No single oxidative marker has achieved sufficient specificity or standardization to function as a stand-alone clinical tool [110,128,129].

#### 5.2.2. Mild Cognitive Impairment (MCI)

MCI is an especially attractive setting for oxidative biomarker research because it lies closer to the window in which early detection might matter clinically. Oxidative DNA damage has been reported in MCI, and urinary or plasma oxidative readouts have been proposed as markers of transition toward AD-spectrum pathology. Even so, the current evidence remains insufficiently consistent to support routine use for early detection. In MCI, oxidative markers are more defensible as risk-enrichment candidates than as validated predictors of conversion [130,131].

#### 5.2.3. Parkinson’s Disease (PD)

In Parkinson’s disease, systematic reviews and meta-analyses support elevations in several oxidative stress markers, including 8-OHdG, MDA, nitrite, and ferritin, with lower levels of some antioxidant measures. These findings support the biological importance of oxidative stress in PD, but they also expose substantial heterogeneity across studies. Accordingly, PD is a compelling yet methodologically unresolved context for oxidative biomarker development: the evidence convincingly supports association, but not yet clinically robust marker performance for diagnosis or longitudinal monitoring [132,133,146].

#### 5.2.4. Dementia with Lewy Bodies (DLB)

Cortical regions from DLB patients have shown increased protein carbonyls and increased oxidized DNA base products, supporting oxidative damage as a measurable component of the disorder in post-mortem material. Broader reviews of dementia biomarkers also discuss approaches to monitoring oxidative stress in the Lewy body dementia spectrum. This supports the idea that oxidative stress is a measurable component of Lewy body spectrum pathology. At the same time, the evidence base remains much smaller than in AD or PD and most data derive from tissue-based or review-level observations rather than mature clinical biomarker pipelines. DLB, therefore, remains biologically convincing but translationally underdeveloped in this domain [147].

#### 5.2.5. Multiple System Atrophy (MSA)

Protein-bound 4-HNE immunoreactivity was localized to neuronal and glial cytoplasmic inclusions in MSA, directly linking a canonical lipid peroxidation aldehyde marker to disease-defining pathology. This makes MSA notable not because oxidative markers are clinically mature, but because the oxidative signal can be tied to a characteristic histopathological substrate. The limitation is obvious; such evidence is strong mechanistically but inherently weak for routine clinical biomarker translation [134].

#### 5.2.6. Progressive Supranuclear Palsy (PSP)

A CSF study quantified Cu/Zn-SOD, GPx, and 4-HNE-conjugated GPx in PSP, explicitly tying tauopathy-spectrum disease to both antioxidant enzyme readouts and lipid peroxidation-linked products [135]. This aligns PSP with the broader neurodegeneration literature, where lipid peroxidation-derived adducts and antioxidant capacity are used as indirect measures of redox burden. However, PSP illustrates a recurring issue in the field: the literature supports mechanistic involvement, yet the biomarker evidence remains too limited and too disease-specific in methodology to justify strong translational claims [1,148].

#### 5.2.7. Corticobasal Degeneration (CBD)

In CBD, oxidative stress responses have been inferred from HO-1 immunoreactivity and related evidence of oxidative injury in tauopathy-associated lesions [136,149]. These findings support the concept that redox dysregulation contributes to pathology in corticobasal degeneration. Nevertheless, the evidence remains largely tissue-based and historical, with limited support from a substantial body of clinically scalable biomarker work. Consequently, CBD should be discussed as a disease in which oxidative stress is biologically implicated, while biomarker development in this area remains at an early stage [150].

#### 5.2.8. Frontotemporal Lobar Degeneration (FTLD/FTD)

Increased 4-HNE adducts were detected in frontal cortex samples in FTLD subtypes, connecting a lipid peroxidation marker to frontotemporal neurodegeneration in human tissue [137]. Oxidative damage in FTLD is therefore biologically plausible and supported by neuropathological evidence. However, as in several atypical neurodegenerative syndromes, the field is still dominated by mechanistic and tissue-based observations rather than clinically deployable biomarker studies [151].

#### 5.2.9. Amyotrophic Lateral Sclerosis (ALS)

A comprehensive review summarizes oxidative stress mechanisms in ALS and discusses how oxidative damage and mitochondrial dysfunction intersect with motor neuron degeneration [138]. Early perspective articles underline the complexity of establishing a direct causal relationship between oxidative stress and ALS, while also documenting substantial research interest in oxidative and nitrosative damage in the disease. Yet the field still faces the same translational limitation seen elsewhere: evidence is stronger for pathogenic relevance than for validated diagnostic or prognostic utility. In ALS, oxidative markers remain promising adjunctive readouts rather than established clinical tools [152].

#### 5.2.10. Huntington’s Disease (HD)

A dedicated review discusses oxidative stress mechanisms in HD and describes the use of oxidative damage markers, including 8-OHdG in human studies such as PREDICT-HD [37]. Longitudinal analysis of leukocyte 8-OHdG has been explored as a progression-related biomarker in HD cohorts. However, the biomarker literature is not fully consistent, because plasma 8-OHdG failed to perform as a biomarker of disease state or progression in other studies. For that reason, current evidence is insufficient to justify routine clinical use, and these markers presently contribute more to biological interpretation than to validated prognostic modeling [139].

#### 5.2.11. Spinocerebellar Ataxia Type 3 (SCA3/MJD)

A human study evaluated ROS production and antioxidant defense capacity in symptomatic and presymptomatic SCA3/MJD individuals, supporting the potential of peripheral redox markers as candidate biomarkers. This finding is important because it suggests that oxidative stress may be detectable outside the CNS in at least some hereditary ataxias. However, the evidence base remains relatively small and translational conclusions should therefore remain cautious [140].

#### 5.2.12. Friedreich’s Ataxia (FRDA)

Urinary 8-OHdG (8-OH2’dG) was reported as increased in FRDA patients, directly linking FRDA to measurable oxidative DNA damage in a clinically accessible sample. Reviews discussing the molecular basis of FRDA highlight how impaired mitochondrial metabolism and iron homeostasis promote oxidative stress and outline potential biomarker approaches, including circulating MDA and urinary 8-OHdG as indicators of systemic oxidative damage. Nevertheless, biomarker coherence does not automatically imply clinical readiness. The main unmet need is longitudinal validation in relation to disease stage and therapeutic response [141].

#### 5.2.13. Multiple Sclerosis (MS)

In MS, oxidative stress biomarkers have been widely reviewed, with emphasis on lipid peroxidation products, oxidative DNA damage, and antioxidant imbalance. This literature supports the view that redox dysregulation is clinically measurable in MS and may relate to disease activity. Yet, as in neurodegeneration more broadly, no single oxidative marker has emerged as sufficiently robust for stand-alone clinical use. In MS, oxidative biomarkers are perhaps best positioned as adjunctive indicators within broader inflammatory and neurodegenerative biomarker panels [142].

#### 5.2.14. Prion Disease/Creutzfeldt–Jakob Disease (CJD)

Prion disease underscores the importance of critically evaluating oxidative stress biomarkers. Classical indicators such as MDA have been tested in both CSF and serum. However, several clinical studies did not demonstrate elevated endogenous levels relative to controls. This does not rule out oxidative involvement in prion disease, but it clearly shows that mechanistic plausibility does not guarantee biomarker positivity or clinical usefulness. CJD is therefore a useful reminder that negative biomarker findings are also informative [143].

#### 5.2.15. Spinobulbar Muscular Atrophy (SBMA)

Cross-sectional and longitudinal analyses support urinary 8-OHdG as a biomarker reflecting severity and potentially predicting motor function deterioration in SBMA [144]. This connects SBMA to the broader neurodegeneration practice of using oxidized nucleic acid products as stable readouts of oxidative stress. Among the rarer disorders discussed here, this is one of the more clinically suggestive oxidative biomarker observations. Even so, replication in larger cohorts remains necessary before strong prognostic claims can be made. The lesson is not that SBMA already has a validated oxidative biomarker, but that this disease may represent a niche setting in which oxidative readouts are closer to practical utility than in many broader neurodegenerative syndromes [33].

#### 5.2.16. Glaucoma (Neurodegenerative Optic Neuropathy)

Glaucoma is worth retaining in this discussion because it expands the neurodegenerative framework beyond classic cortical or extrapyramidal disorders. Systematic review and meta-analysis support increased oxidative and antioxidative stress marker abnormalities in chronic glaucoma, including frequent use of MDA and changes in antioxidant status. This supports the inclusion of glaucoma as a neurodegenerative condition in which oxidative burden is clinically quantifiable. At the same time, the same limitation persists: marker abnormalities support pathogenic relevance, but not disease specificity [145].

## 6. Current and Emerging Therapeutic Strategies Targeting Mitochondrial Function

Therapeutic strategies targeting mitochondrial dysfunction in neurodegenerative and primary mitochondrial disorders have progressively shifted from broad antioxidant supplementation toward mechanism-informed interventions that aim to modulate mitochondrial quality control, bioenergetic adaptation, organelle dynamics, mitochondrial genome maintenance, targeted delivery, and intercellular mitochondrial rescue. This shift reflects a central lesson from translational studies: although mitochondrial impairment is a recurrent feature of neurodegenerative diseases, it is not always the primary driver of pathology. Therefore, the probability of therapeutic success is likely to be highest when mitochondrial dysfunction is proximal to disease biology, measurable through pathway-relevant biomarkers and targeted in biologically enriched patient populations [1].

### 6.1. Antioxidant Strategies

Antioxidant-based interventions were among the earliest mitochondria-oriented therapeutic approaches, based on the contribution of impaired electron transport, oxidative and nitrosative stress, lipid peroxidation, and maladaptive redox signaling to neuronal injury. However, clinical experience in common sporadic neurodegenerative disorders has failed to support antioxidant reinforcement as a treatment strategy [148]. MitoQ, a mitochondria-targeted ubiquinone derivative designed to accumulate within mitochondria, did not slow disease progression in a double-blind, placebo-controlled trial in newly diagnosed Parkinson’s disease [153]. Similarly, high-dose coenzyme Q10 failed to show clinical benefit in the phase III QE3 trial in early Parkinson’s disease, despite a strong mechanistic rationale related to electron transport and redox homeostasis [35]. Inosine-mediated urate elevation also illustrates this translational limitation. In the SURE-PD3 trial, inosine increased serum urate but did not reduce the rate of clinical progression in early Parkinson’s disease [34]. Idebenone should be interpreted as a disease-context-specific exception rather than as validation of antioxidant therapy across neurodegeneration. Its strongest relevance is in Leber hereditary optic neuropathy, where respiratory chain dysfunction is genetically anchored and mechanistically proximal to disease biology. In the LEROS nonrandomized controlled trial, idebenone showed treatment benefit in LHON, with effects influenced by disease phase and the causative mtDNA mutation. This distinction is important because it suggests that redox-active mitochondrial therapies may be more effective in genetically defined mitochondrial disorders than in heterogeneous neurodegenerative disorders [36]. These studies support the biological relevance of oxidative mitochondrial injury, but they provide limited evidence that antioxidant reinforcement alone can modify disease progression in common neurodegenerative disorders.

### 6.2. Mitochondrial Biogenesis Enhancers and NAD+-Directed Therapies

A second therapeutic approach aims to enhance mitochondrial resilience by modulating adaptive metabolism, mitochondrial biogenesis, and NAD+-dependent signaling pathways. This approach is broader than conventional antioxidant therapy because it seeks to improve mitochondrial and metabolic maintenance rather than only neutralize reactive species. However, the strongest human evidence supports target engagement rather than established disease modification [154]. Among NAD+ precursors, nicotinamide riboside has one of the strongest translational foundations in Parkinson’s disease. In the randomized, double-blind phase I NADPARK trial, nicotinamide riboside was well tolerated and produced measurable changes in brain metabolism. These findings demonstrate that NAD+-directed pharmacology can engage the human brain, but they do not establish clinical efficacy [37]. Resveratrol provides a related example in Alzheimer’s disease. In a randomized, placebo-controlled trial, high-dose resveratrol was safe and well tolerated and altered selected biomarker trajectories, but it did not establish clear clinical benefit [38]. Therefore, NAD+-linked and biogenesis-oriented strategies are best described as proof-of-biology approaches whose disease-modifying potential remains to be determined in larger trials.

### 6.3. Mitophagy Modulation

Mitophagy modulation is one of the most mechanistically compelling areas in mitochondrial therapeutics because it targets organelle quality control rather than downstream metabolic consequences. The therapeutic aim is not simply to increase mitochondrial turnover, but to selectively enhance the removal of dysfunctional mitochondria while preserving the mitochondrial pool required for neuronal function [155]. USP30 is currently among the most promising targets in this field. In an α-synuclein-driven Parkinson’s disease mouse model, genetic deletion or pharmacological inhibition of USP30 protected dopaminergic neurons, reduced pathological α-synuclein-related changes, and supported mitophagy-related mechanisms. These findings provide strong preclinical support, but they should not be interpreted as clinical evidence of efficacy in humans [39]. PINK1- and Parkin-directed pharmacology requires particular caution. Recent work indicates that putative activators such as MTK458 and related compounds may lower the threshold for mitophagy by sensitizing cells to mitochondrial stress, rather than acting as clean pathway agonists. This distinction is important because nonspecific mitochondrial stress could have unintended consequences in neurons, where mitochondrial turnover must remain tightly regulated [156]. Mechanism-linked pharmacodynamic biomarkers may strengthen the translational development of this class. Phospho-Ser65 ubiquitin is not a therapeutic intervention but rather a pathway-proximal readout of PINK1/Parkin activity and mitochondrial damage. ELISA-based detection of pS65-Ub in human cells, autopsy brain tissue, and blood samples may therefore support proof-of-mechanism studies for mitophagy-directed therapies [157].

### 6.4. Modulators of Mitochondrial Dynamics

Mitochondrial dynamics are essential for neuronal homeostasis. Therapeutic modulation of this system is challenging because mitochondrial fission is not inherently pathological; it is required for mitochondrial redistribution, mitophagy, and adaptive remodeling. A clinically useful strategy would therefore need to suppress maladaptive fragmentation without disabling physiological mitochondrial remodeling [158]. P110 was designed to interfere with the pathological DRP1-FIS1 interaction and has been shown in preclinical systems to reduce excessive mitochondrial fragmentation while relatively sparing other DRP1-dependent interactions. Its selectivity makes it conceptually more attractive than broad fission inhibition, although its evidence base remains predominantly preclinical [159]. By contrast, mdivi-1 should be discussed as a cautionary example rather than as a therapeutic candidate. Although it was widely used as a DRP1 inhibitor, later work showed that mdivi-1 can act predominantly as a reversible inhibitor of mitochondrial complex I-dependent respiration and reverse electron transport-associated reactive oxygen species production at commonly used experimental concentrations. These findings substantially complicate the interpretation of earlier studies and illustrate the importance of rigorous chemical validation in mitochondrial pharmacology [160].

### 6.5. Metabolic Modulators

Metabolic modulators aim to improve mitochondrial function by altering substrate use, supporting anaplerosis, stabilizing mitochondrial membranes, or engaging adaptive metabolic signaling. These approaches are attractive because pharmacodynamic effects can sometimes be assessed using metabolic imaging, spectroscopy, or biochemical endpoints. However, clinical efficacy has been inconsistent [161]. Ursodeoxycholic acid is best described as a mechanistically plausible mitochondrial stabilizer rather than an established disease-modifying therapy. In a double-blind, randomized phase II trial in early Parkinson’s disease, high-dose UDCA was safe and well tolerated, and the use of phosphorus magnetic resonance spectroscopy provided an organelle-relevant pharmacodynamic dimension. Nevertheless, larger trials are required to determine whether these biological effects translate into clinically meaningful benefit [162]. The fixed-dose combination of sodium phenylbutyrate and taurursodiol (PB-TURSO; AMX0035) illustrates the fragility of early translational signals in mitochondrial and endoplasmic-reticulum stress-directed therapy. The CENTAUR phase II trial in amyotrophic lateral sclerosis reported slower functional decline over 24 weeks [163]. However, the larger 48-week PHOENIX phase III trial missed both its primary and secondary endpoints, and the product was subsequently voluntarily withdrawn from the US and Canadian markets in October 2024, supporting a cautious interpretation of AMX0035 as an example of early translational promise requiring confirmatory validation [164]. Triheptanoin provides an inconclusive example. Its anaplerotic rationale is coherent, but randomized phase II data in early Huntington’s disease showed no slowing of caudate atrophy compared with placebo over six months. Longer-term observations and external-control comparisons may be hypothesis-generating, but they are not sufficient to establish disease modification [165]. Therefore, metabolic modulation remains biologically informative, but current evidence more consistently supports pharmacodynamic engagement than reproducible clinical efficacy.

### 6.6. Mitochondrial Gene Therapy, mtDNA Editing and Mitochondrial Protein Replacement

Therapies that address mitochondrial dysfunction at a more causal molecular level are among the fastest-developing areas of the field. These include allotopic gene expression, heteroplasmy shifting, mitochondrial genome editing and mitochondrial protein replacement. Their shared rationale is that correcting an upstream mitochondrial defect may be more effective than compensating for downstream oxidative or metabolic consequences. However, delivery specificity, tissue tropism, immunogenicity, dose control, and off-target effects remain major translational barriers [166]. Lenadogene nolparvovec is one of the most clinically advanced mitochondria-relevant gene therapy approaches. In MT-ND4-related Leber hereditary optic neuropathy (LHON), five-year follow-up showed sustained bilateral improvement in best-corrected visual acuity and a favorable long-term safety profile after unilateral intravitreal administration. Although this is a highly specific disease context, it demonstrates that gene therapy directed at mitochondrial disease biology can reach sustained clinical evaluation [167]. Mitochondrial-targeted nucleases, including mitoTALENs, extend this concept by selectively reducing mutant mtDNA and shifting heteroplasmy. In vivo work has shown that mitoTALENs can reduce mutant mtDNA load in central nervous system neurons, supporting the feasibility of neuronal mitochondrial genome engineering [154]. DdCBE base editing further expands the field by enabling nucleotide-level mitochondrial DNA editing, but recent mouse data also define a critical safety bottleneck: high-dose AAV9-DdCBE can induce extensive off-target mitochondrial editing and severe adverse effects. These findings indicate that editing efficiency must be balanced against dose-dependent toxicity and off-target risk [168]. Nomlabofusp represents a related but distinct strategy. Rather than editing mtDNA, it is designed as a frataxin replacement therapy for Friedreich’s ataxia. Phase I data showed favorable short-term safety, pharmacokinetic characterization, and pharmacodynamic evidence of increased frataxin in accessible tissues, making it a useful human example of mitochondrial restoration pharmacology. Even with these advances, significant challenges remain unresolved. Mitochondrial base editors act on a multicopy genome in which therapeutic advantage requires shifting heteroplasmy past a tissue-specific threshold, and bystander editing, off-target deamination of nuclear DNA, and durable delivery to postmitotic neurons all remain unsolved. Currently, no mtDNA-editing approach has entered clinical testing in a neurodegenerative indication, and none should presently be regarded as clinically viable [169].

### 6.7. Mitochondrial Transplantation

Mitochondrial transplantation involves the delivery of viable mitochondria to injured tissue with the aim of restoring bioenergetic capacity and limiting secondary injury. At present, its strongest rationale lies in acute injury settings rather than chronic neurodegenerative disease. The way of administration is one of the principal problems, and intranasal delivery has been proposed as a way around it: transport along olfactory and trigeminal pathways bypasses the blood–brain barrier, and preclinical studies report uptake of intranasally administered mitochondria into brain tissue with functional benefit. The approach remains at an early stage, and the delivered dose reaching defined neuronal populations has not been quantified, where repeated dosing, biodistribution, neuronal uptake, long-term integration, and scalability are more difficult to control. Recent work has begun to address the targeting problem directly: a cell-type-specific mitochondrial delivery system based on engineered protein binders achieved selective uptake into defined neuronal and non-neuronal populations, improved respiration and survival of patient-derived neurons carrying an MT-ND4 mutation in vitro, and increased retinal ganglion cell survival after optic nerve injury in vivo [40]. Human experience remains at an early feasibility stage [170]. A phase I report of autologous mitochondrial transplantation for acute cerebral ischemia during mechanical thrombectomy described the standardized isolation of viable autologous mitochondria in the acute clinical setting and reported no major adverse effects from delivery via blood vessels. This should be framed as a platform-establishing feasibility study, not as evidence of clinical efficacy. Current limitations include mitochondrial isolation protocols, organelle viability, potency assays, biodistribution, scalability, and reproducibility across clinical settings [171].

### 6.8. Inner-Membrane and Cardiolipin-Targeted Small Molecules and Peptides

A distinct therapeutic strategy targets the inner mitochondrial membrane, cardiolipin-dependent cristae architecture, and respiratory-chain organization. This approach is attractive because it addresses structural determinants of mitochondrial efficiency rather than only downstream oxidative damage [172]. Elamipretide is the leading translational example in this category. By interacting with cardiolipin-rich mitochondrial membranes, it is intended to stabilize cristae organization and improve respiratory efficiency. However, in the MMPOWER-3 randomized clinical trial in primary mitochondrial myopathy, elamipretide was well tolerated but did not improve the six-minute walk distance or fatigue after 24 weeks compared with placebo. This trial is important because it shows that mechanistic coherence does not necessarily translate into clinical efficacy, especially in heterogeneous mitochondrial disease populations [173].

### 6.9. Nanoparticle and Mitochondria-Targeted Delivery Platforms

Delivery technologies should be considered enabling platforms rather than stand-alone therapies. Their purpose is to overcome one of the major challenges in mitochondrial medicine, delivering therapeutic cargo to the correct tissue, cell type, intracellular compartment, and mitochondrial subcompartments without unacceptable toxicity [41]. Triphenylphosphonium remains a canonical mitochondria-targeting motif because lipophilic cations can accumulate in mitochondria in a membrane-potential-dependent manner. However, TPP^+^ is a physicochemical targeting principle rather than a therapy, and its usefulness depends on cargo chemistry, dosing, tissue distribution, and mitochondrial membrane potential [174]. MITO-Porter provides a complementary proof-of-concept platform for direct mitochondrial cargo delivery via membrane fusion, including macromolecular delivery into mitochondria [175]. More recent work has extended MITO-Porter-based systems to mitochondrial delivery of CRISPR/Cas9 ribonucleoproteins [176]. Mitochondria-penetrating peptides are similarly important as organelle-access tools, but they remain delivery systems rather than mature therapeutic entities [177]. These approaches are important because they may enable future mitochondrial therapeutics, but their translational value will depend on whether they improve in vivo delivery without compromising safety, specificity or scalability. The barriers are considerable and largely unaddressed for this class. Cationic mitochondria-targeting moieties, such as triphenylphosphonium, cross the blood–brain barrier poorly, and systemically administered carriers are predominantly cleared by the liver and spleen, so the fraction reaching the brain parenchyma is small. Uptake within the brain is further biased toward microglia and endothelium rather than the neurons of interest, and intact organelles are far larger than any payload for which these platforms were optimized. Quantitative biodistribution and cell-type-resolved uptake data, rather than demonstrations of mitochondrial colocalization in culture, are the evidence this field currently lacks.

### 6.10. Cell Therapies and Mitochondrial Transfer

**Figure 4:**
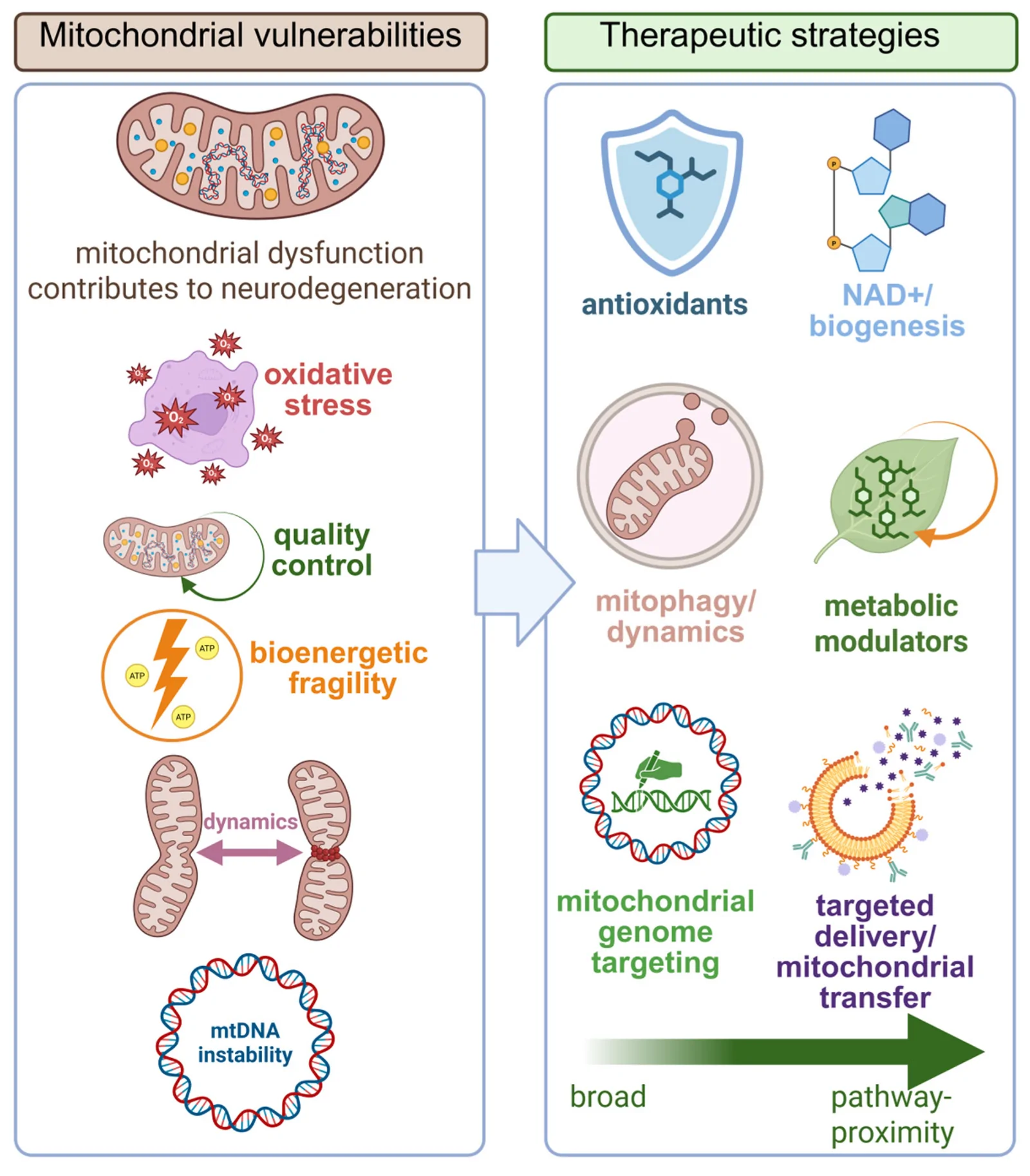
Mitochondrial modulation as a therapeutic entry point in neurodegeneration. (**Left**), mitochondrial processes whose failure links organelle dysfunction to neuronal vulnerability: oxidative stress, loss of quality control, bioenergetic fragility, disturbed fusion–fission dynamics and mitochondrial DNA instability. (**Right**), therapeutic classes reviewed in Section 4, ordered by mechanistic proximity to mitochondrial biology (arrow): broad antioxidants, NAD^+^ augmentation and biogenesis, mitophagy and dynamics modulation, metabolic modulators, mitochondrial genome targeting, and platforms for targeted delivery and mitochondrial transfer. Most rest on proof-of-mechanism rather than proof-of-efficacy evidence; the strongest translational signals arise where mitochondrial dysfunction is genetically anchored. mtDNA, mitochondrial DNA; NAD^+^, nicotinamide adenine dinucleotide. Created in BioRender. Kulbacka, J. (2026) (https://BioRender.com/6rp5rgd).

Cell-based approaches intersect with mitochondrial therapeutics in two distinct ways. First, restorative cell therapies may replace vulnerable or lost neuronal populations and thereby improve circuit-level function. Second, intercellular mitochondrial transfer may be exploited as a rescue mechanism for bioenergetically compromised cells. These approaches should not be conflated with conventional mitochondria-targeted drugs, but they are relevant to the broader therapeutic strategy [178]. Recent dopaminergic progenitor transplantation trials provide important human evidence for regenerative strategies in Parkinson’s disease. A phase I/II trial of allogeneic iPSC-derived dopaminergic progenitors showed graft survival, dopamine production, absence of tumor formation, and acceptable early safety [179]. A parallel phase I trial of hES-derived dopaminergic progenitors reported early safety and biological activity after putaminal grafting. These studies are highly relevant to regenerative medicine, but they should be clearly distinguished from direct mitochondria-targeted interventions [180]. For the mitochondrial component specifically, engineered mitochondria-enriched extracellular vesicles provide an important preclinical example. These vesicles aim to improve the quantity and quality of transferable mitochondrial material, thereby optimizing a process that can occur through extracellular vesicles and other intercellular routes [181]. Broader work on mesenchymal stromal cell-mediated mitochondrial transfer highlights tunneling nanotubes, extracellular vesicles, and related mechanisms, while also emphasizing unresolved issues of efficiency, standardization, potency testing, and therapeutic reproducibility [182]. This subsection should therefore be framed as a frontier area linking regenerative medicine, intercellular organelle transfer and mitochondrial rescue, rather than as a mature class of mitochondria-targeted therapies. The principal mitochondria-directed therapeutic strategies are organized according to their mechanistic proximity and translational maturity in Figure 4.

The translational success of mitochondria-targeted interventions will likely depend not only on the intervention itself but also on mechanism-informed trial design. Neurodegenerative diseases are biologically heterogeneous, and patients may differ substantially in the timing, magnitude, and cellular localization of mitochondrial dysfunction. Biomarkers of oxidative injury, including lipid peroxidation products such as malondialdehyde, 4-HNE, and F2-isoprostanes, protein oxidation markers such as protein carbonyls, and oxidative DNA damage markers such as 8-OHdG, may support pharmacodynamic monitoring and biological stratification, although their specificity and reproducibility vary across disease contexts. F2-isoprostanes and related neuroprostanes are widely discussed as oxidative stress biomarkers in neurodegenerative diseases, whereas pS65-Ub is more closely associated with the PINK1/Parkin-dependent mitophagy pathway [157,183]. Current evidence indicates that mitochondrial dysfunction is a compelling therapeutic target, but not a uniformly druggable driver across neurodegenerative diseases. Broad antioxidant reinforcement has largely failed to modify disease progression in sporadic Parkinson’s disease, whereas more mechanism-informed strategies, including NAD+ augmentation, mitophagy modulation, metabolic rewiring, mitochondrial genome engineering, targeted delivery, and mitochondrial transfer, show varying degrees of biological plausibility and pharmacodynamic engagement. The strongest translational rationale emerges when mitochondrial dysfunction is genetically anchored, pathway-proximal, and measurable, as illustrated by selected inherited mitochondrial disorders. In contrast, most approaches in common neurodegenerative diseases remain limited by biological heterogeneity, incomplete target validation, insufficient patient stratification, and a frequent gap between early biomarker effects and durable clinical benefit. Future studies will require molecularly enriched cohorts, pathway-relevant pharmacodynamic biomarkers and clinically meaningful endpoints to determine whether mitochondrial therapeutics can progress from mechanistic plausibility to reproducible disease modification. Table 3 below summarizes these strategies by evidentiary maturity and indicates the disease contexts in which each is most defensible. Read together, the table makes the central argument of this review concrete: the classes with genuine clinical traction are those addressing a genetically anchored, pathway-proximal lesion, whereas broadly acting interventions have repeatedly failed in sporadic disease.

## 7. Mitochondria-Targeted Therapies as Complementary Approaches to Conventional Neurodegenerative Disease Treatments

Mitochondria-targeted therapies are not likely to replace established treatments for major neurodegenerative diseases anytime soon. A more practical approach is to consider them complementary to treatments that mainly focus on neurotransmission, protein clumping, or gene-related issues. This perspective is backed by the fact that while mitochondrial dysfunction is rarely the only cause, it significantly affects neuronal vulnerability, synaptic failure, disrupted protein balance, and the reduced ability to handle chronic stress. In this context, mitochondrial medicine may be most valuable in strengthening cellular resilience after primary disease treatment rather than serving as a standalone therapy. Practically, this implies add-on designs in which a mitochondria-directed agent is layered onto an established backbone, such as anti-amyloid antibodies for Alzheimer’s disease, dopaminergic replacement for Parkinson’s disease, or riluzole for amyotrophic lateral sclerosis. Such trials should enroll biomarker- or genotype-selected patients, be powered for delayed functional decline rather than short-term symptomatic gain, and include a pathway-proximal pharmacodynamic readout to confirm target engagement before efficacy is judged [2,3].

**Figure 5:**
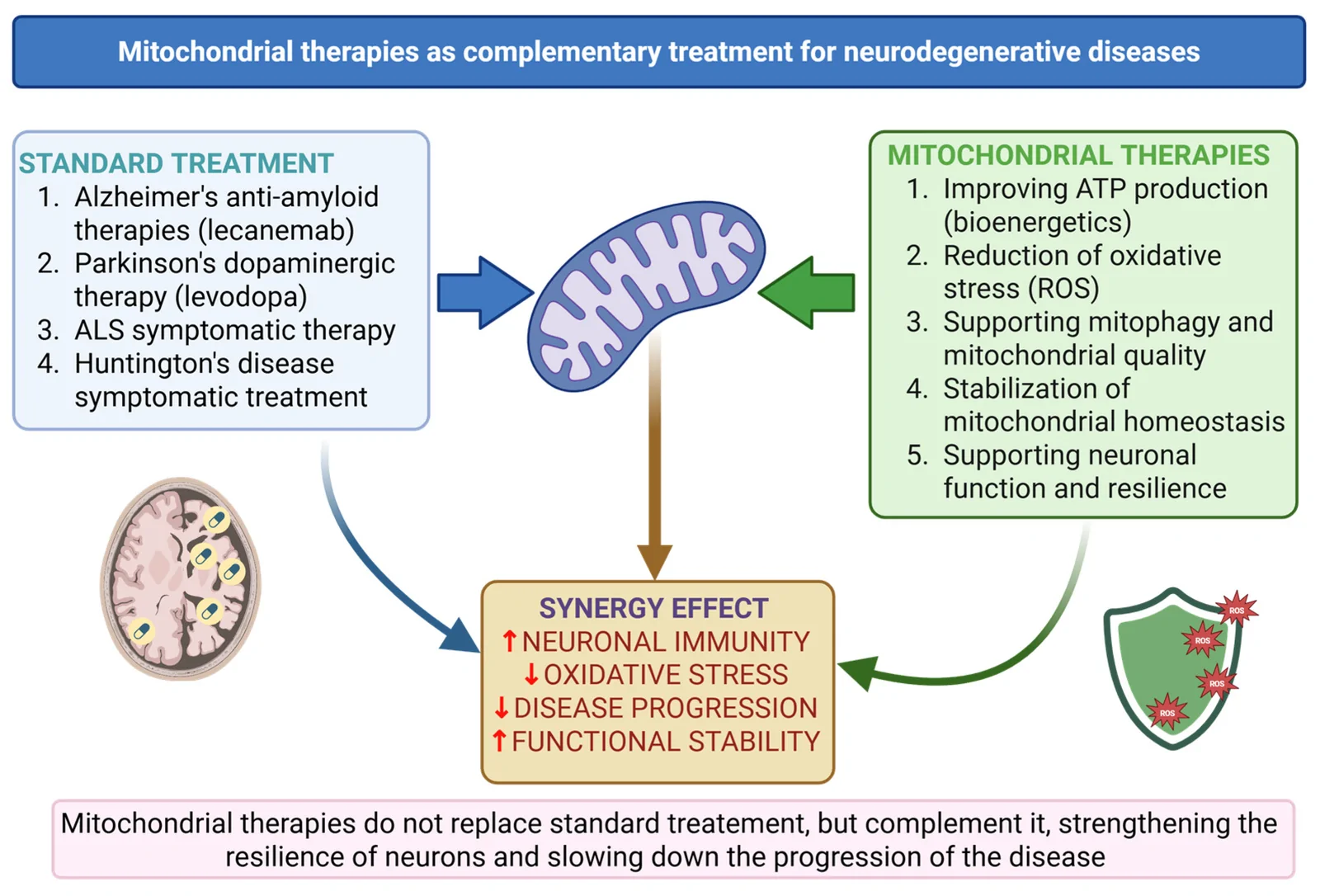
Mitochondrial therapies as complementary treatment for neurodegenerative diseases. Established disease-specific treatments—anti-amyloid antibodies in Alzheimer’s disease, dopaminergic replacement in Parkinson’s disease, and largely symptomatic management in amyotrophic lateral sclerosis and Huntington’s disease—address the primary pathology but do not restore bioenergetic capacity, redox balance, or organelle quality control. Mitochondria-targeted agents are therefore positioned as add-on interventions intended to enhance neuronal resilience in biomarker-selected patients, with benefit assessed by delayed functional decline, biomarker stability, and reduced need for treatment escalation rather than by short-term symptomatic improvement. Blue arrows indicate the contribution of standard treatment and green arrows the contribution of mitochondrial therapies; both converge on mitochondrial function and on the resulting synergy. The brown arrow indicates the combined outcome of both treatment arms. Within the synergy box, upward arrows denote an increase and downward arrows a decrease in the indicated parameter. Created in BioRender. Kulbacka, J. (2026) (https://BioRender.com/6rp5rgd).

This complementary approach is particularly important in Alzheimer’s disease. Anti-amyloid antibodies like lecanemab and donanemab have demonstrated that removing amyloid can slow clinical decline in early symptomatic cases, but these treatments do not directly restore mitochondrial bioenergetics, correct redox imbalance, or improve organelle quality. Therefore, the next logical step is not to position mitochondria-focused therapies against amyloid-lowering antibodies but to see if they can stabilize neuronal function once the primary issues have been partially managed. Mitochondrial interventions may be most beneficial as enhancers of disease-modifying therapy, especially in biologically selected patients who remain metabolically vulnerable despite confirmed target engagement [184,185,186]. A similar argument holds for Parkinson’s disease, though the treatment setting is different. Symptomatic dopaminergic therapy is crucial to current management, with levodopa remaining central for motor symptoms. However, symptomatic treatment does not address mitochondrial quality control, oxidative stress, or the energy fragility of dopaminergic neurons. For this reason, mitochondria-targeted strategies in Parkinson’s disease should be seen as additions to symptomatic therapy. Practically, this suggests that real benefits might be better tracked through delayed functional decline, stabilized biomarkers, or reduced need for treatment escalation, rather than by short-term symptom improvement over standard agents [187,188,189]. The adjunctive model may be even more relevant for amyotrophic lateral sclerosis, where current therapies offer limited benefit and progression is rapid despite treatment. In ALS, mitochondrial dysfunction connects with excitotoxicity, RNA issues, axonal transport failure, and neuroinflammation. This makes mitochondria-targeted therapies appealing not as separate alternatives to standard treatment but as stabilizing additions that may help sustain motor neuron function while other therapies target different aspects of the disease. This principle can also be cautiously applied to Huntington’s disease, where current management is primarily symptomatic, and even a small boost to resilience could be clinically significant if initiated early and combined with optimized support [187,189,190]. Mitochondria-targeted therapies are more likely to be effective when combined with disease stage enrichment, relevant biomarkers, and endpoints that can capture biological stability rather than just short-term symptom improvement. In practice, this means identifying patients with measurable mitochondrial vulnerability, integrating pharmacodynamic markers of target engagement, and assessing these therapies alongside established care standards rather than against them. Under this approach, mitochondrial medicine shifts from seeking a universal standalone treatment to creating a precise platform for adjunct neuroprotection. This shift represents both a more realistic clinical outlook and a more innovative conceptual approach than the expectation that mitochondrial modulation alone will significantly alter the course of complex neurodegenerative diseases [157,184]. The proposed role of mitochondria-targeted therapies as adjuncts to disease-specific treatment is illustrated in Figure 5.

## 8. Conclusions

Mitochondria are no longer viewed only as passive producers of cellular energy, but as active regulators of neuronal development, synaptic function, calcium signaling, redox balance, organelle quality control, and neuroinflammatory responses. This broad biological influence makes mitochondrial dysfunction a recurring feature of neurodegenerative diseases and an attractive therapeutic target. However, mitochondrial impairment is neither a uniform mechanism across neurodegenerative disorders nor universally druggable. A major conclusion emerging from current research is that the therapeutic value of mitochondrial modulation depends strongly on disease context. Broad antioxidant strategies have generally failed to produce convincing clinical benefit in common sporadic neurodegenerative diseases, suggesting that simply reducing oxidative stress is not sufficient to restore complex neuronal homeostasis. More targeted approaches, including NAD+ augmentation, mitophagy modulation, regulation of mitochondrial dynamics, metabolic interventions, mitochondrial genome engineering, targeted delivery systems, and mitochondrial transfer, offer more precise mechanistic opportunities. Nevertheless, many of these strategies remain limited by incomplete target validation, biological heterogeneity, insufficient patient stratification, and a persistent gap between biomarker engagement and durable clinical improvement. Therefore, the future of mitochondrial medicine in neurodegeneration will likely depend on precision-based application rather than universal therapeutic use. The strongest potential may lie in genetically defined mitochondrial disorders or in biomarker-enriched subgroups of patients in whom mitochondrial dysfunction is measurable, pathway-proximal and clinically relevant. In more complex disorders, such as Alzheimer’s disease, Parkinson’s disease, amyotrophic lateral sclerosis or Huntington’s disease, mitochondria-targeted interventions may be most effective as adjunctive therapies that enhance neuronal resilience and complement established disease-specific treatments. Future studies should prioritize mechanism-informed trial design, molecularly selected cohorts, pathway-relevant pharmacodynamic biomarkers, and endpoints capable of measuring long-term neuronal stabilization rather than only short-term symptomatic change. Under this framework, mitochondrial modulation represents a promising but carefully delimited therapeutic entry point, not a universal solution for neurodegeneration, but a biologically rational strategy to strengthen vulnerable neurons when applied in the right disease stage, patient population, and therapeutic context.

## Figures and Tables

**Table 2:** Free radical–related biomarkers across neurodegenerative diseases.

Disease	Oxidative Biomarkers	Sample Types	Main Translational Signal	Main Limitation
Alzheimer’s disease (AD)	MDA, 4-HNE, F2-isoprostanes, 8-OHdG/8-oxodG	Brain, CSF, blood, urine	One of the strongest disease contexts for oxidative biomarker research, with repeated association with lipid peroxidation and oxidative DNA injury	Strong association, but limited specificity and poor stand-alone diagnostic performance [128,129]
Mild cognitive impairment (MCI)	8-OHdG/8-oxodG, MDA	Urine, blood/plasma	Biologically attractive pre-dementia setting closer to the putative early-detection window	Current evidence is insufficiently consistent for routine prediction of conversion [130,131]
Parkinson’s disease (PD)	8-OHdG, MDA, nitrite, ferritin, reduced antioxidant measures in some studies	Blood, CSF, brain	Repeated evidence of oxidative stress across meta-analyses supports biological relevance	Substantial inter-study heterogeneity, clinical utility remains unresolved [132,133]
Dementia with Lewy bodies (DLB)	Protein carbonyls, oxidized DNA base products	Post-mortem cortex, limited clinical matrices	Oxidative injury is detectable in cortical tissue and fits Lewy body spectrum pathology	Evidence base is much smaller than for AD/PD and remains largely tissue-based [117]
Multiple system atrophy (MSA)	Protein-bound 4-HNE in neuronal and glial cytoplasmic inclusions	Post-mortem brain	Strong histopathological linkage between lipid peroxidation and disease-defining inclusions	Pathology-linked but not clinically scalable [134]
Progressive supranuclear palsy (PSP)	4-HNE-conjugated GPx, antioxidant enzyme changes	CSF	Supports redox involvement in tauopathy-spectrum disease	Evidence remains limited and methodologically narrow [135]
Corticobasal degeneration (CBD)	HO-1 immunoreactivity, oxidative stress-related lesion signals	Post-mortem brain	Suggests oxidative stress response in tauopathy-associated lesions	Largely historical, tissue-based literature, minimal clinical biomarker development [136]
Frontotemporal lobar degeneration (FTLD/FTD)	4-HNE adducts	Post-mortem frontal cortex	Supports lipid peroxidation involvement in frontotemporal neurodegeneration	Dominated by tissue-based observations rather than scalable clinical studies [137]
Amyotrophic lateral sclerosis (ALS)	Urinary 8-oxodG, urinary/plasma isoprostanes, broader oxidative stress signatures	Urine, plasma, tissue	Strong mechanistic overlap with mitochondrial dysfunction, axonal injury and proteostasis failure	Evidence remains stronger for pathogenic relevance than for validated prognostic utility [138]
Huntington’s disease (HD)	8-OHdG/8-oxodG	Blood/leukocytes, plasma	Oxidative DNA damage is biologically plausible and has been explored longitudinally	Evidence is mixed; leukocyte and plasma readouts have not been consistently concordant [139]
Spinocerebellar ataxia type 3 (SCA3/MJD)	Peripheral ROS elevation, reduced antioxidant defense	Blood	Suggests measurable peripheral oxidative phenotype in hereditary ataxia	Small evidence base and limited replication [140]
Friedreich’s ataxia (FRDA)	Urinary 8-OHdG	Urine	Mechanistically coherent because mitochondrial dysfunction and iron dysregulation are central to disease biology	Longitudinal validation remains limited [141]
Multiple sclerosis (MS)	MDA/4-HNE, 8-OHdG, 8-iso-PGF2α, antioxidant imbalance	CSF, blood	Oxidative stress is repeatedly measurable and may relate to disease activity	No single marker is sufficiently robust for stand-alone use [142]
Prion disease/CJD	Classical lipid peroxidation markers such as MDA have been tested	CSF, serum	Useful as a negative or mixed example showing that oxidative plausibility does not guarantee marker positivity	At least some studies failed to show elevated endogenous MDA versus controls [143]
Spinobulbar muscular atrophy (SBMA/Kennedy disease)	Urinary 8-OHdG	Urine	One of the more clinically suggestive oxidative biomarker signals among rarer disorders; linked to severity and later decline	Requires replication in larger cohorts [144]
Glaucoma (neurodegenerative optic neuropathy)	MDA, AOPP, 8-OHdG, antioxidant status changes	Serum, ocular fluids	Supports inclusion of glaucoma within the broader neurodegenerative oxidative-stress framework	Pathogenic relevance exceeds specificity [145]

**Table 3:** Mitochondria-directed therapeutic strategies ordered by evidentiary maturity. Strategies are listed in the order discussed in Section 6. “Clinical” denotes completed randomized trials in humans; “preclinical” denotes cell or animal evidence only. FRDA, Friedreich ataxia; LHON, Leber hereditary optic neuropathy; PD, Parkinson’s disease; MSC, mesenchymal stromal cell; TPP^+^, triphenylphosphonium.

Strategy (Section)	Representative Agents	Mechanism	Evidentiary Maturity	Best-Suited Context
Antioxidants (Section 6.1)	MitoQ, coenzyme Q10, idebenone	Scavenging of reactive species; electron-carrier support	Clinical, largely negative in sporadic disease (QE3, MitoQ in PD); idebenone controlled evidence in LHON	Genetically defined mitochondrial disease, not sporadic neurodegeneration
NAD^+^ and biogenesis (Section 6.2)	Nicotinamide riboside, bezafibrate	PGC-1α-driven biogenesis; NAD^+^-dependent signaling	Clinical, target engagement demonstrated but disease modification unproven	Early PD; settings with a measurable NAD^+^ pharmacodynamic readout
Mitophagy modulation (Section 6.3)	USP30 inhibitors, MTK458	Selective clearance of damaged organelles	Preclinical; pS65-Ub available as pathway-proximal biomarker	PINK1- or PRKN-mutant PD
Dynamics modulation (Section 6.4)	P110; mdivi-1 as cautionary example	Suppression of maladaptive DRP1-FIS1 fission	Preclinical; mdivi-1 confounded by off-target complex I inhibition	Disorders with documented excess fission
Metabolic modulators (Section 6.5)	Ursodeoxycholic acid, PB-TURSO	Substrate use, anaplerosis, membrane stabilization	Clinical, inconsistent; PHOENIX negative and product withdrawn	Contexts with an imaging or spectroscopy pharmacodynamic endpoint
Genome targeting and protein replacement (Section 6.6)	Allotopic expression, mtDNA base editors, nomlabofusp	Correction of the primary genetic lesion	Clinical for LHON and FRDA; editing entirely preclinical	LHON, FRDA and other monogenic mitochondrial disease
Transplantation (Section 6.7)	Isolated mitochondria; targeted delivery systems	Replacement of dysfunctional organelles	Early feasibility; strongest rationale in acute injury	Optic neuropathy; acute rather than chronic disease
Cardiolipin-targeted agents (Section 6.8)	Elamipretide (SS-31)	Cristae stabilization; respiratory-chain organization	Clinical, MMPOWER-3 negative for primary endpoint	Primary mitochondrial myopathy
Delivery platforms (Section 6.9)	TPP^+^ conjugates, targeted nanoparticles	Organelle-directed delivery	Preclinical; enabling platform rather than therapy	Cross-cutting; limited by CNS biodistribution
Cell therapy and transfer (Section 6.10)	Dopaminergic progenitors; MSC-mediated transfer	Cell replacement; intercellular organelle rescue	Phase I/II for cell replacement; transfer preclinical	PD for cell replacement; transfer mechanistic only

## Data Availability

No new data were created or analyzed in this study. Data sharing is not applicable to this article. Grammarly was used for language editing.

## Abbreviations

The following abbreviations are used in this manuscript:

2D	Two-dimensional
3-NT	3-Nitrotyrosine
4-HNE	4-Hydroxy-2-nonenal
8-iso-PGF2α	8-Iso-prostaglandin F2α
8-OHdG	8-Hydroxy-2′-deoxyguanosine
8-oxodG	8-Oxo-2′-deoxyguanosine
53BP1	p53-Binding protein 1
AAV9	Adeno-associated virus serotype 9
AD	Alzheimer’s disease
AGEs	Advanced glycation end-products
ALS	Amyotrophic lateral sclerosis
AOPP/AOPPs	Advanced oxidation protein product/products
ApoB-100	Apolipoprotein B-100
ATP	Adenosine triphosphate
CAT	Catalase
CBD	Corticobasal degeneration
CD/CDs	Conjugated diene/dienes
CJD	Creutzfeldt–Jakob disease
CNS	Central nervous system
CoA	Coenzyme A
CRISPR/Cas9	Clustered regularly interspaced short palindromic repeats/CRISPR-associated protein 9
CSF	Cerebrospinal fluid
Cu/Zn-SOD	Copper/zinc superoxide dismutase
DdCBE	DddA-derived cytosine base editor
DLB	Dementia with Lewy bodies
DNA	Deoxyribonucleic acid
DNPH	2,4-Dinitrophenylhydrazine
DPC/DPCs	DNA–protein crosslink/crosslinks
DRP1	Dynamin-related protein 1
DSB/DSBs	Double-strand break/breaks
E3	E3 ubiquitin ligase
ECD	Electrochemical detection
ELISA	Enzyme-linked immunosorbent assay
EM	Electron microscopy
EPR	Electron paramagnetic resonance
ER	Endoplasmic reticulum
ESI	Electrospray ionization
Fapy	Formamidopyrimidine
Fapy-dA	Adenine-derived formamidopyrimidine lesion
Fapy-dG	Guanine-derived formamidopyrimidine lesion
FIS1	Mitochondrial fission 1 protein
FITC	Fluorescein isothiocyanate
FOX	Ferrous oxidation–xylenol orange
FRDA	Friedreich’s ataxia
FTD	Frontotemporal dementia
FTLD	Frontotemporal lobar degeneration
GBA1	Glucosylceramidase beta 1
GC	Gas chromatography
GC-MS	Gas chromatography–mass spectrometry
GDAP1	Ganglioside-induced differentiation-associated protein 1
GPx	Glutathione peroxidase
GSH-Px	Glutathione peroxidase
GTPase	Guanosine triphosphatase
H3K27ac	Acetylation of lysine 27 on histone H3
H4K16ac	Acetylation of lysine 16 on histone H4
HD	Huntington’s disease
HDAC	Histone deacetylase
hES	Human embryonic stem cell
HO-1	Heme oxygenase 1
HPLC	High-performance liquid chromatography
HPLC-ECD	High-performance liquid chromatography with electrochemical detection
HPLC-ESI-MS/MS	High-performance liquid chromatography–electrospray ionization tandem mass spectrometry
ID	Identification
IL-1β	Interleukin-1 beta
IL-18	Interleukin-18
iPSC/iPSCs	Induced pluripotent stem cell/cells
LC-MS/MS	Liquid chromatography–tandem mass spectrometry
LDL	Low-density lipoprotein
LHON	Leber hereditary optic neuropathy
LOOH	Lipid hydroperoxide/hydroperoxides
mAb	Monoclonal antibody
MAM/MAMs	Mitochondria-associated membrane/membranes
MAPK	Mitogen-activated protein kinase
MCI	Mild cognitive impairment
MDA	Malondialdehyde
mdivi-1	Mitochondrial division inhibitor 1
MDV/MDVs	Mitochondria-derived vesicle/vesicles
MERCS	Mitochondria–endoplasmic reticulum contact sites
MFN2	Mitofusin 2
miR-124	MicroRNA-124
MITO-Porter	Mitochondrial delivery liposome-based carrier system
mitoTALEN/mitoTALENs	Mitochondria-targeted transcription activator-like effector nuclease/nucleases
MitoQ	Mitochondria-targeted ubiquinone
MJD	Machado–Joseph disease
MnSOD	Manganese superoxide dismutase
MRC	Mitochondrial respiratory chain
MS	Multiple sclerosis or mass spectrometry, depending on context
MS/MS	Tandem mass spectrometry
MSA	Multiple system atrophy
MT-ND4	Mitochondrially encoded NADH dehydrogenase subunit 4
mtDAMP/mtDAMPs	Mitochondrial damage-associated molecular pattern/patterns
mtDNA	Mitochondrial DNA
NAD^+^	Oxidized nicotinamide adenine dinucleotide
NADH	Reduced nicotinamide adenine dinucleotide
NF-κB	Nuclear factor kappa B
NLRP3	NLR family pyrin domain-containing 3
NOX	Nicotinamide adenine dinucleotide phosphate oxidase
NRF-1	Nuclear respiratory factor 1
Nrf2	Nuclear factor erythroid 2-related factor 2
NSC/NSCs	Neural stem cell/cells
OPA1	Optic atrophy protein 1
OXPHOS	Oxidative phosphorylation
oxLDL	Oxidized low-density lipoprotein
PB-TURSO	Sodium phenylbutyrate–taurursodiol
PC/PCs	Protein carbonyl/carbonyls
PD	Parkinson’s disease
PET	Positron emission tomography
PGC-1α	Peroxisome proliferator-activated receptor gamma coactivator 1-alpha
PI3K	Phosphoinositide 3-kinase
PINK1	PTEN-induced kinase 1
POLG	DNA polymerase gamma catalytic subunit
PSP	Progressive supranuclear palsy
pS65-Ub	Serine-65-phosphorylated ubiquitin
PTPIP51	Protein tyrosine phosphatase-interacting protein 51
PUFA/PUFAs	Polyunsaturated fatty acid/acids
RAB7/Rab7	Ras-related protein Rab-7
RNA	Ribonucleic acid
RNS	Reactive nitrogen species
ROS	Reactive oxygen species
SBMA	Spinal and bulbar muscular atrophy
SCA3	Spinocerebellar ataxia type 3
SDS	Sodium dodecyl sulfate
SKN	Student Scientific Society
SLC25A32	Solute carrier family 25 member 32
SOD	Superoxide dismutase
SSB/SSBs	Single-strand break/breaks
STRIDE	SensiTive Recognition of Individual DNA Ends
TBA	Thiobarbituric acid
TBARS	Thiobarbituric acid-reactive substance
TBC1D15	TBC1 domain family member 15
TFAM	Mitochondrial transcription factor A
Tg	Thymidine glycol
TNF	Tumor necrosis factor
TPP^+^	Triphenylphosphonium cation
TUNEL	Terminal deoxynucleotidyl transferase dUTP nick-end labeling
TWNK	Twinkle mitochondrial DNA helicase
UDCA	Ursodeoxycholic acid
UHPLC	Ultra-high-performance liquid chromatography
UPLC	Ultra-performance liquid chromatography
US	United States
USP30	Ubiquitin-specific peptidase 30
UV	Ultraviolet
VAPB	Vesicle-associated membrane protein-associated protein B
XRCC1	X-ray repair cross-complementing protein 1
Ca^2+^	Calcium ion
K^+^	Potassium ion
Na^+^	Sodium ion
ΔΨm	Mitochondrial membrane potential
γH2AX	Histone H2AX phosphorylated at serine 139
